# Ischemia-reperfusion injury: molecular mechanisms and therapeutic targets

**DOI:** 10.1038/s41392-023-01688-x

**Published:** 2024-01-08

**Authors:** Meng Zhang, Qian Liu, Hui Meng, Hongxia Duan, Xin Liu, Jian Wu, Fei Gao, Shijun Wang, Rubin Tan, Jinxiang Yuan

**Affiliations:** 1 The Collaborative Innovation Center, Jining Medical University, Jining, Shandong 272067 China; 2 Clinical Medical College, Jining Medical University, Jining, Shandong 272067 China; 3 Second Clinical Medical College, Jining Medical University, Jining, Shandong 272067 China; 4 Shanghai Institute of Cardiovascular Diseases, Zhongshan Hospital and Institutes of Biomedical Sciences, Fudan University, Shanghai, China; 5 Institute of Zoology, Chinese Academy of Sciences, Beijing, China; 6 Department of Physiology, Basic medical school, Xuzhou Medical University, Xuzhou, 221004 China

**Keywords:** Cardiology, Molecular biology, Cardiovascular diseases

## Abstract

Ischemia-reperfusion (I/R) injury paradoxically occurs during reperfusion following ischemia, exacerbating the initial tissue damage. The limited understanding of the intricate mechanisms underlying I/R injury hinders the development of effective therapeutic interventions. The Wnt signaling pathway exhibits extensive crosstalk with various other pathways, forming a network system of signaling pathways involved in I/R injury. This review article elucidates the underlying mechanisms involved in Wnt signaling, as well as the complex interplay between Wnt and other pathways, including Notch, phosphatidylinositol 3-kinase/protein kinase B, transforming growth factor-β, nuclear factor kappa, bone morphogenetic protein, N-methyl-D-aspartic acid receptor-Ca^2+^-Activin A, Hippo-Yes-associated protein, toll-like receptor 4/toll-interleukine-1 receptor domain-containing adapter-inducing interferon-β, and hepatocyte growth factor/mesenchymal-epithelial transition factor. In particular, we delve into their respective contributions to key pathological processes, including apoptosis, the inflammatory response, oxidative stress, extracellular matrix remodeling, angiogenesis, cell hypertrophy, fibrosis, ferroptosis, neurogenesis, and blood-brain barrier damage during I/R injury. Our comprehensive analysis of the mechanisms involved in Wnt signaling during I/R reveals that activation of the canonical Wnt pathway promotes organ recovery, while activation of the non-canonical Wnt pathways exacerbates injury. Moreover, we explore novel therapeutic approaches based on these mechanistic findings, incorporating evidence from animal experiments, current standards, and clinical trials. The objective of this review is to provide deeper insights into the roles of Wnt and its crosstalk signaling pathways in I/R-mediated processes and organ dysfunction, to facilitate the development of innovative therapeutic agents for I/R injury.

## Introduction

**Fig. 1:**
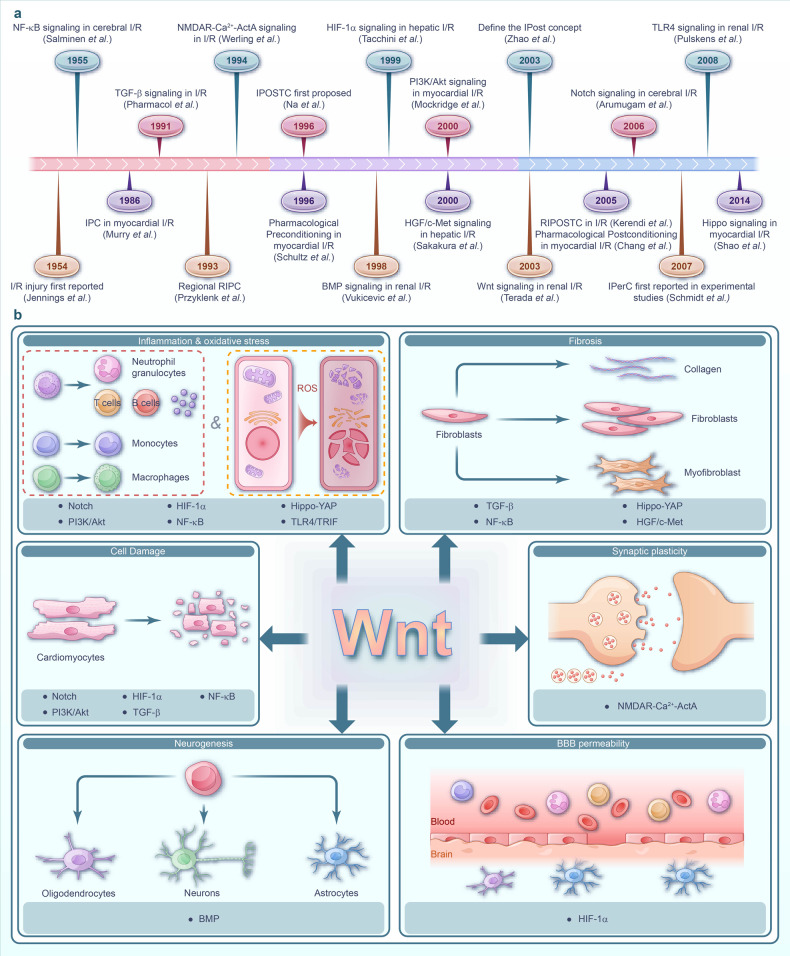
The intricate signaling network in I/R injury pathogenesis. **a** Timeline diagram of key milestones in I/R Injury research: revealing crucial discoveries and emphasizing complex signaling pathways. **b** The complexity of signaling pathways in I/R injury Pathology. Involvement of Wnt signaling and crosstalk with diverse signaling pathways in the pathological process of I/R injury: impact on cellular injury, inflammation, oxidation, fibrosis, neurogenesis, synaptic plasticity, and BBB permeability across organs I/R events. I/R ischemia-reperfusion, NF-κB nuclear factor-κB, IPC ischemic preconditioning, TGF-β transforming growth factor-β, RIPC remote ischemic preconditioning, NMDAR N-Methyl-D-Aspartate Receptor, ActA Activin A, IPOSTC ischemic postconditioning, BMP bone morphogenetic protein, HIF-1α hypoxia-inducible factor-1α, PI3K/Akt phosphoinositide-3 kinase/protein kinase B, HGF/c-Met hepatocyte growth factor receptor/mesenchymal-epithelial transition factor, RIPOSTC remote ischemic postconditioning, YAP Yes-associated protein, TLR4/TRIF toll-like receptor 4/toll-interleukine-1 receptor domain-containing adapter-inducing interferon-β, BBB blood-brain barrier

Ischemia of organs can have severe consequences such as myocardial infarction (MI) and cerebral infarction, leading to irreversible tissue damage.^1,2^ Tissue reperfusion is employed to prevent further ischemia; however, in some cases, it may worsen the injury through a process known as ischemia-reperfusion (I/R) injury,^3,4^ which can occur in many organs and result in additional disorders, disability, and even death.^5^ Multiple pathological processes are involved in I/R injuries, such as cell damage (apoptosis, necrosis, and ferroptosis), oxidative stress, inflammatory response, blood-brain barrier (BBB) breakdown, extracellular matrix (ECM) remodeling, angiogenesis, cardiomyocyte hypertrophy, and fibrosis.^6–13^ Extensive research has been dedicated to unraveling the mechanisms and therapeutic strategies associated with signaling pathways implicated in I/R injury. Several key pathways including Notch, phosphatidylinositol 3-kinase/protein kinase B (PI3K/Akt), transforming growth factor-β (TGF-β), nuclear factor kappa (NF-κB), bone morphogenetic protein (BMP), N-methyl-D-aspartic acid receptor (NMDAR)-Ca^2+^-Activin A, hippo-yes-associated protein (YAP), toll-like receptor 4/toll-interleukine-1 receptor domain-containing adapter-inducing interferon-β (TLR4/TRIF) and hepatocyte growth factor/mesenchymal-epithelial transition factor (HGF/c-Met), and Wnt, have emerged as crucial players in this context.^14–24^ Fig. 1a depicts the research milestones in the exploration of signaling pathways during I/R injury. Among these pathways, the Wnt signaling pathway, which has attracted attention, consists of multiple branches, with the canonical Wnt/β-catenin, non-canonical Wnt/PCP and Wnt/Ca^2+^ pathways being particular important for I/R injury. Evidence suggests that different branches of the Wnt pathway play distinct roles in various pathological processes.^10,25–29^ The Wnt pathway interacts with various key signaling pathways, creating an extensive network that collectively regulates I/R injury as shown in Fig. 1b. During and through I/R injury, the Wnt pathway interacts with NF-κB or HIF-1α signaling, thereby regulating inflammation and oxidative stress responses.^30,31^ Additionally, the crosstalk between the Wnt pathway and other signaling pathways, including Notch, PI3K/Akt, TGF-β, and NF-κB, is implicated in the regulation of apoptosis.^32,33^ Moreover, the Wnt/BMP signaling crosstalk is involved in regulating neurogenesis,^34^ while direct interaction between NMDAR-Ca^2+^-ActA and Wnt signaling modulates synaptic plasticity.^35–41^ Furthermore, the Wnt pathway crosstalk with Hippo-YAP, TGF-β, HGF/c-Met, NF-κB, and other signaling pathways regulate fibrosis in organs like the heart, kidney, and liver following I/R injury, which can potentially lead to adverse outcomes.^35–44^ The current treatment strategies include pre-ischemic preconditioning, post-ischemic preconditioning, and medicine preconditioning^45–48^ (Fig. 1a). However, the intricate complexity of I/R injury, along with the interconnections among various signaling pathways, remains significantly challenging. Therefore, there remains a lack of consensus in current research,^38–43^ which limits the advancement of treatment strategies. This article provides a comprehensive overview of the intricate interplay between Wnt signaling and other signaling pathways in the complex signaling network involved in I/R injury (Fig. 1b). The evidence encompasses studies conducted on patients, as well as findings from various animal and cell models. Additionally, by elucidating the underlying mechanisms, we outline current clinical and preclinical therapeutic strategies that target the Wnt pathway and interconnected signaling pathway networks (Fig. 1a). Considering the complex nature of organ damage in I/R injury, targeting network signaling pathways is crucial for effective interventions. Future studies should focus on developing strategies that effectively modulate these interconnected signals to mitigate the detrimental effects of I/R damage.

## Wnt pathways and organ I/R Injury

### Wnt pathways

The Wnt signaling pathway is an essential regulator^49^ involved in various cell activities, including proliferation, differentiation, migration, and development^50–52^ and consists of Wnt ligand proteins, Wnt receptors, and other signal transduction accessories such as scattered (Disheveled, Dsh/Dvl) proteins. The pathway can be divided into two categories base on its dependence on β-catenin, namely canonical and non-canonical pathways. Currently, 19 different Wnt ligand proteins have been identified, with some predominantly activating the canonical pathway (Wnt1, Wnt2, Wnt3, Wnt3a, Wnt8a, Wnt8b, Wnt10a, and Wnt10b) and others primarily activating the non-canonical pathway (Wnt4, Wnt5a, Wnt5b, Wnt6, Wnt7a, Wnt7b, and Wnt11).^51,53^ However, evidence suggests that some ligands (such as Wnt3a, Wnt5a, and Wnt9b) function in both the canonical and non-canonical Wnt pathways.^53,54^ Frizzled proteins serve as the primary receptors for Wnt signals,^55^ and function in conjunction with co-receptors such as low-density lipoprotein receptor-related protein 5/6 (LRP5/6)^56^ and tyrosine kinase co-receptors like recombinant receptor tyrosine kinase (RYK) like orphan receptor 1/2 (ROR1/2) and RYK.^57^ In the absence of Wnt ligands, β-catenin is targeted for degradation by a “destruction complex” consisting of Axin, adenomatous polyposis coli protein, Casein kinase 1α, and glucogen synthase kinase 3β (GSK-3β).^58^ However, in the presence of the Wnt ligands, the Wnt/β-catenin pathway is activated through binding the ligands to Frizzleds receptor and co-receptor LRP5/6. Then, β-catenin accumulates in the cytoplasm and is translocated to the nucleus where it binds to the T-cell factor and lymphoid enhancer factor (TCF/LEF),^32,33,42–44^ initiating the transcription of Wnt downstream target genes.^59,60^ Conversely, the non-canonical Wnt signaling pathway operates independently of β-catenin and includes the Wnt/planar cell polarity (PCP) and Wnt/Ca^2+^ pathways,^61^ both of which are activated when Wnt ligands bind to Frizzleds protein and ROR1/2.^49,62–65^ In the Wnt/PCP pathway, the activation of small G proteins Rho or Rac1 triggers the activation of c-Jun N-terminal kinase (JNK), which in turn plays a critical role in the rearrangement of the actin cytoskeleton and regulating cell polarity and promoting migration.^51,66–69^ In the Wnt/Ca^2+^ pathway, activated PLC induces IP3 production, leading to a substantial increase in intracellular Ca^2+^ levels. This triggers the activation of Ca^2+^-dependent effector molecules, including calmodulin-dependent protein kinase II (CaMKII), protein kinase-C (PKC), and calcineurin, and the nuclear factor of activated T cells (NFAT) to initiate the transcription of genes associated with Ca^2+^-related signaling.^49,60,70–72^

### Wnt pathways during myocardial I/R injury

In the treatment of heart and vascular diseases, such as atherosclerosis, coronary artery disease, MI, arrhythmia, myocardial hypertrophy, and heart failure, timely myocardial reperfusion through thrombolysis or percutaneous coronary intervention therapy is crucial.^73–76^ It can salvage viable myocardium, limit the extent of MI, preserve left ventricular systolic function, and prevent heart failure,^77^ during which I/R injury often occurs, leading to various detrimental effects on the heart.^73–76^ The Wnt signaling pathway, initially involved in early heart development and typically inactive under normal conditions, plays a significant role in cardiovascular diseases.^61^ In the context of myocardial I/R injury, the Wnt pathway is engaged in various I/R-associated processes, including apoptosis,^78^ inflammatory responses, oxidative stress, ECM remodeling, angiogenesis, cardiac hypertrophy, and fibrosis.^4,79–89^ Various cell types within the heart, such as cardiac precursor cells, cardiomyocytes, fibroblasts, endothelial cells (ECs), epicardium, smooth muscle cells, adipocytes, and macrophages,^85,86,90^ play key roles in heart injury via cell-to-cell communication, in which the Wnt signaling pathway serve as a central regulator.^85,91,92^ With the increasing prevalence of cardiovascular diseases due to population aging, addressing the elevated risk of organ damage resulting from myocardial I/R is of utmost importance.

#### Apoptosis

**Fig. 2:**
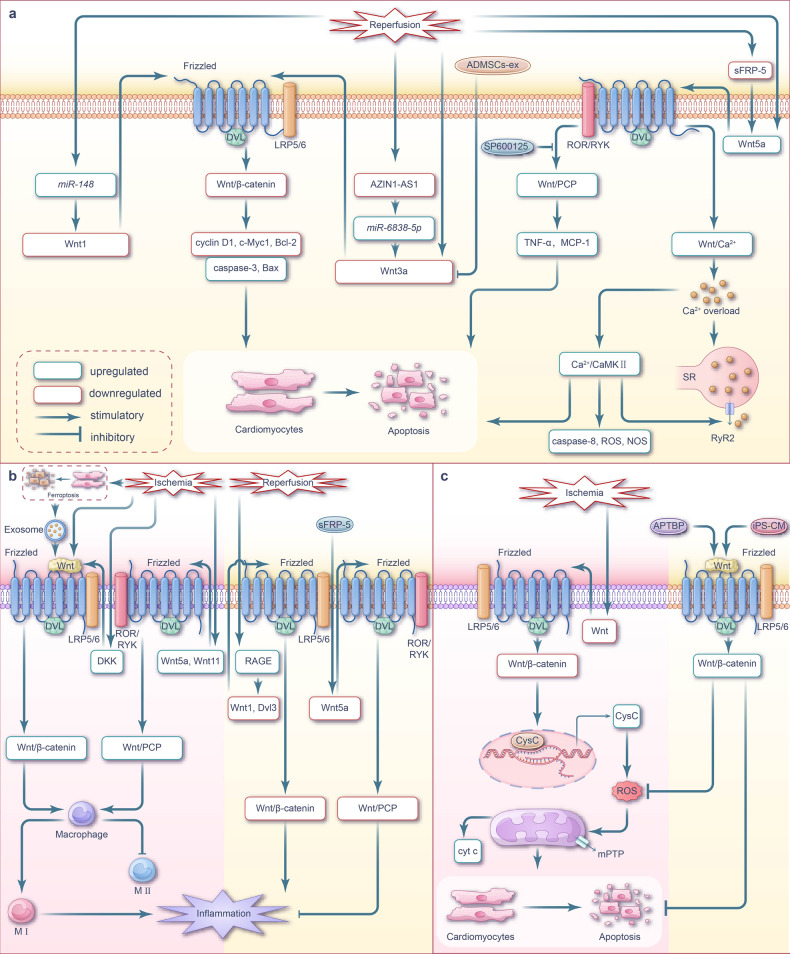
Wnt signaling pathway and targeted therapy for apoptosis, inflammation, and oxidative stress during myocardial I/R injury. **a** Wnt signaling pathway-mediated apoptosis during myocardial I/R injury. During myocardial I/R injury, the inhibition of Wnt/β-catenin signaling promotes cardiomyocytes apoptosis. However, the roles of non-canonical Wnt/PCP and Wnt/Ca^2+^ signaling pathways in myocardial I/R injury are the opposite. The activation of these two Wnt signaling pathways may exacerbate cardiomyocyte apoptosis through the activation of the JNK pathway or the induction of calcium overload. **b** Wnt signaling-mediated inflammation during myocardial I/R injury. Cardiomyocyte ferroptosis occurs during the ischemia phase, and the exosomes derived from the ferroptotic cells induce M1 macrophage transformation by activating the Wnt/β-catenin pathway. During myocardial ischemic phase, the activation of Wnt/β-catenin signaling promotes the polarization of macrophages towards the M1 phenotype while suppressing the M2 phenotype, ultimately exacerbating the inflammatory response. Upregulation of Wnt ligands and DKK family members in macrophages stimulates inflammation by activating the Wnt/β-catenin signaling pathway. During the process I/R injury, there is an upregulation of RAGE expression in the infarct border zone of rat cardiomyocytes, accompanied by downregulation of Wnt1 and Dvl3 expression. By inhibiting the Wnt/β-catenin signaling pathway, this leads to the promotion of inflammatory response, exacerbating cardiomyocyte apoptosis. Additionally, activation of the Wnt/PCP pathway in macrophages during the ischemic phase increases the expression of inflammatory cytokines, which aggravates cardiac inflammation. **c** Wnt signaling-mediated oxidative stress during myocardial I/R injury. During myocardial ischemia phase, the downregulation of Wnt protein inhibits Wnt/β-catenin signaling transduction, resulting in increased transcription of intracellular CysC. The elevated expression of CysC exacerbates intracellular oxidative stress and promotes the generation of ROS leading to cardiomyocyte apoptosis. Note: The pink background represents the ischemic phase, while the cream background represents the reperfusion phase. ADMSCs-ex exosomes isolated from adipose-derived mesenchymal stem cells, sFRP-5 secretory frizzled-related protein 5, LRP low-density lipoprotein receptor-related protein, ROR recombinant receptor tyrosine kinase like orphan receptor, RYK receptor tyrosine kinase, CaMKII calmodulin-dependent protein kinase II, ROS reactive oxygen species, NOS nitric oxide synthase, RyR ryanodine receptors, TNF-α tumor necrosis factor-α, MCP-1 monocyte chemoattractant protein-1, APTBP a peptide from tuna backbone protein, CysC cystatin C, mPTP mitochondrial permeability transition pore, cyt c cytochrome c, iPS-CM induced plenipotentiary stem cell-derived conditioned medium

Myocardial I/R inhibits Wnt/β-catenin signaling and upregulates apoptosis; meanwhile, Wnt/PCP and Wnt/Ca^2+^ signaling pathways have also been implicated in apoptosis activation (Fig. 2a).

In the myocardium of I/R-induced rats and hypoxia/reoxygenation (H/R)-induced H9C2 cells, a significant upregulation of *miR-148b* was observed, which inhibited the Wnt/β-catenin signaling by downregulating Wnt1 expression. Consequently, the expression of β-catenin, cyclin D1, C-myc, and the ratio of Bcl-2/Bax was downregulated, while the ratio of cleaved caspase 3 and p-GSK-3β/GSK-3β was upregulated. These changes ultimately increased ischemic area and cardiomyocyte apoptosis rate in I/R rats.^93^ Zhang et al. found that AZIN1-AS1 was significantly downregulated and *miR-6838-5p* was significantly upregulated in the myocardium of myocardial I/R rats and H/R H9C2 cells; this dysregulation ultimately led to the downregulation of Wnt3a expression, which in turn inhibited Wnt/β-catenin signaling and induced apoptosis.^94^ Additionally, Cui et al. established that the inhibition of Wnt/β-catenin signaling in I/R rat myocardium and H/R H9c2 cells, initiated by the downregulation of Wnt3a, promoted cardiomyocyte apoptosis.^95^

Within the non-canonical Wnt signaling pathway,^96,97^ secreted frizzled-related protein 5 (sFRP-5) acts as an extracellular inhibitor that counteracts Wnt5a-mediated signaling pathway.^98,99^ JNK, an essential component of Wnt/PCP signaling, is activated via the non-canonical Wnt pathway.^98,100,101^ Following I/R treatment, the downregulation of sFRP-5 transcription in the pericardial fat of mice activated macrophage in the injured heart. This activation subsequently upregulated Wnt5a expression, increased JNK phosphorylation, and elevated the expression levels of inflammatory cytokines IL-1β and TNF-α, as well as the chemokine MCP1, which ultimately promoted cardiomyocyte apoptosis via Wnt/PCP pathway activation.^102^

Zhou et al. demonstrated that the protein levels of Wnt5a and Frizzled2 were elevated, along with the increased intracellular calcium concentration in the myocardium of cardiac I/R rats and H/R H9C2 cells.^103^ It has been postulated that the Wnt/Ca^2+^ pathway mediates Ca^2+^ accumulation and promotes apoptosis. Indeed, Ca^2+^ overload during myocardial I/R triggers the production of caspase 8, oxygen free radicals, and nitric oxide, which alters the redox environment of calcium channel proteins and transporters. Therefore, the Wnt-associated Ca^2+^ channels, known as ryanodine receptors, undergo a series of changes during myocardial I/R, including redox modification, phosphorylation, and nitrosation, thereby inducing the dysfunctional opening of diastolic ryanodine receptors channels, leading to ventricular remodeling, arrhythmia, and untimely heart failure.^104–106^

These findings suggest that targeting the upstream molecules of the Wnt signaling pathway or Wnt itself can inhibit apoptosis and ameliorate myocardial injury in I/R by reversing Wnt signaling (Fig. 2a). For instance, targeting *miR-148b*^93^ or *miR-6838-5p*,^94^ or adding Wnt3a protein^78^ before hypoxia has been reported to inhibit cardiomyocyte apoptosis by upregulating Wnt/β-catenin signaling. Cui et al. utilized adipose-derived mesenchymal stem cell exosomes (ADMSC-ex) to treat myocardial I/R. These exosomes upregulated Wnt3a, p-GSK-3β (Ser9), and β-catenin, activated Wnt/β-catenin signaling, upregulated Bcl-2 and cyclin D1, inhibited Bax expression and caspase3 activity, antagonized I/R-induced cardiomyocyte apoptosis, and increased the cell survival rates.^95^ Alternatively, treatment with recombinant sFRP-5 protein and the JNK inhibitor SP600125 has been reported to inhibit apoptosis by downregulating Wnt/PCP signaling.^102^

#### Inflammatory response

An intense surge in cell death over a short period can trigger an inflammatory response and activate cell repair–related pathways. Inflammation serves as an adaptive cellular response to injury. Immune inflammatory pathways play an important role in cardiac injury and repair. However, excessive inflammatory responses can cause severe and irreversible damage to cardiomyocytes.^107^ The Wnt signaling pathway has emerged as a key regulator of inflammatory responses in myocardial injury, particularly in acute myocardial infarction (AMI) models. Previous studies have shown that myocardial I/R activates Wnt/β-catenin signaling to promote inflammatory responses; however, contrasting studies have reported that inhibition of Wnt/β-catenin signaling promotes such responses. Nonetheless, there is also evidence that non-canonical Wnt signal activation promotes inflammatory responses^61^ (Fig. 2b).

Following MI, macrophages play distinct roles in left ventricular remodeling. Macrophage polarization and classification are critical for their diverse roles in immune function. An imbalance between pro-inflammatory macrophage (M1) and anti-inflammatory macrophage (M2) activities reflects the inflammatory state of the local cardiac tissue microenvironment.^108,109^ “In MI stage phase 1 (inflammatory phase,^110–112^ i.e., 1-4 days after infarction^110–113^), the macrophages recruited in the infarct area are predominantly M1 type, which secrete pro-inflammatory factors such as TNFα, IL1β, IL6, IL10 to remove cell debris; In the stage phase 2 of MI (reparative phase,^110–112^ i.e., the 5 -7days after infarction), M2 macrophages are predominantly recruited in the infarct area. The transformation of macrophages helps to promote the regression of inflammation and the repair of damaged myocardium.^113,114^ However, persistent induction of macrophage M1 phenotype polarization aggravates the inflammatory response through the secretion of IFN-γ,^115^ leading to cardiomyocyte apoptosis and ECM degradation.^116^, thereby aggravating myocardial injury.”

**Fig. 3:**
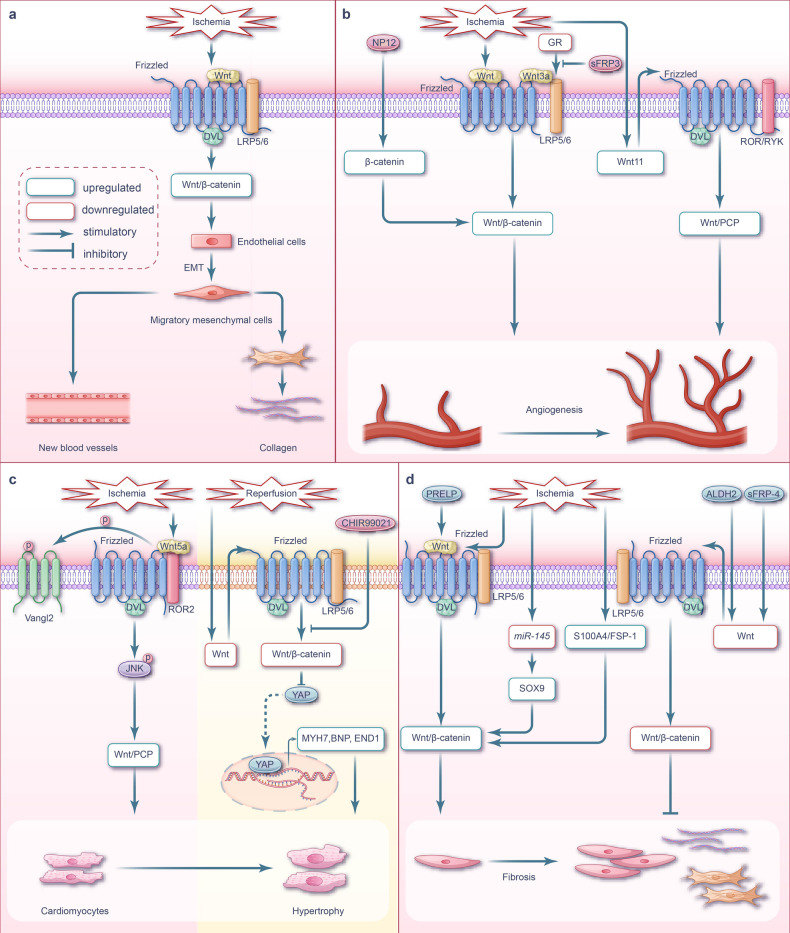
Wnt signaling pathway and targeted therapy in ECM remodeling, angiogenesis, cardiac hypertrophy, and fibrosis during myocardial I/R injury. **a** Wnt signaling-mediated ECM remodeling during myocardial I/R injury. During myocardial ischemic phase, there is a substantial activation of Wnt/β-catenin in endothelial cells, which leads to the promotion of EMT. **b** Wnt signaling-mediated angiogenesis during myocardial I/R injury. During myocardial ischemic phase, Wnt/β-catenin signaling is activated in vascular endothelial cells, which affects the proliferation and migration of these cells during the process of neovascularization. Conversely, in the infarct region of ischemic mice, the activation of the canonical Wnt pathway functions to suppress cardiac angiogenesis. NP12, an allosteric inhibitor of GSK-3β promotes cardiac angiogenesis by activating Wnt/β-catenin signaling. Additionally, the deficiency of GR activates Wnt/β-catenin signaling, leading to the upregulation of cyclin D1 and ultimately promoting cardiac angiogenesis. Moreover, Wnt/PCP activation contributes to angiogenesis during myocardial ischemia injury. **c** Wnt signaling pathway-mediated cardiac hypertrophy during myocardial I/R injury. During myocardial ischemic phase, Wnt5a triggers the activation of Wnt/PCP signaling through JNK phosphorylation, which subsequently induces induces cardiomyocyte hypertrophy. In the AC16 human cardiomyocyte cell line model of I/R injury, Wnt/β-catenin signaling is inhibited, and this inhibition synergistically exacerbates myocardial hypertrophy in cooperates with YAP signaling. **d** Wnt signaling-mediated fibrosis during myocardial I/R injury. During myocardial ischemic phase, the expression of Wnt1 is increased, resulting in the activation of Wnt/β-catenin signaling in cardiac fibroblasts. This activation leads to the proliferation of fibroblasts and ultimately contributes to cardiac repair. *MiR‐145* expression was lower in myocardial ischemic phase. The down-regulation of *miR-145* directly targets SOX9 in fibroblasts, leading to cardiac fibrosis by activating Wnt/β-catenin signaling. Both PRELP and S100A4/FSP1 promote myocardial fibrosis by activating Wnt/β-catenin signaling. On the other hand, the Wnt antagonist sFRP-4 reduces myocardial fibrosis by inhibiting Wnt/β-catenin signaling. Additionally, ALDH2 suppresses myocardial fibrosis by inhibiting Wnt/β-catenin signaling. Note: The pink background represents the ischemic phase, while the cream background represents the reperfusion phase. EMT endothelial-mesenchymal transition, GR glucocorticoid receptor, sFRP secretory frizzled-related protein, LRP low-density lipoprotein receptor-related protein, ROR recombinant receptor tyrosine kinase like orphan receptor, RYK receptor tyrosine kinase, YAP Yes-associated protein, MYH7 myosin heavy chain 7, BNP brain natriuretic peptide, END1 endothelin 1, SOX9 sex‐determining region Y box 9, FSP1 fibroblast-specific protein 1, PRELP proline/arginine-terminal leucine-rich repeat protein

Further, both canonical and non-canonical Wnt signaling pathways can promote the polarization of macrophages toward the M1 phenotype and inhibit M2 phenotype polarization.^117,118^ Therefore, Wnt signaling activation in cardiomyocytes following ischemia may induce cell death in a macrophage-dependent manner, ultimately aggravating myocardial injury (Fig. 3b). Wnt/β-catenin signaling is activated by the inflammatory response during ischemia.^119^ Sun et al. demonstrated that the malondialdehyde content and Fe^2+^ concentration in the hearts of mice significantly increased following MI, while the expression of ischemia-susceptibility marker NOS2 was upregulated and that of the M2-polarization marker IL-10 was downregulated in macrophages. The authors suggested that cardiomyocyte ferroptosis occurs during the ischemia phase, accompanied by macrophage polarization toward the M1 phenotype and verified that hypoxic HL-1 cells undergo ferroptosis in vitro and the exosomes derived from ferroptotic HL-1 cells induce M1 macrophage transformation in RAW264.7 cells by activating the Wnt/β-catenin pathway.^120^

Using a mouse ischemia model, a previous study has demonstrated that β-catenin-mediated signaling was activated in cardiac macrophages, especially in pro-inflammatory subsets.^121^ In vitro experiments confirmed that β-catenin activation, and its associated signal transduction pathway, exhibited pro-inflammatory activity in the mouse macrophage cell line RAW264.7 when transduced by lentivirus.^121^

The expression of Wnt ligands and DKK family members is significantly upregulated in macrophages following MI.^122^ β-Catenin activation could potentially be attributed to this increase in Wnt ligand expression, although the precise factors involved in this process are yet to be determined. The expression of receptors associated with advanced glycation end products is associated with cell migration, proliferation, inflammation, and I/R injury.^123,124^ Park et al. determined that RAGE was upregulated, while Wnt1 and Dvl3 were downregulated at the infarct edge of rat I/R cardiomyocytes, consequently promoting the inflammatory response and exacerbating cardiomyocyte apoptosis by inhibiting the Wnt/β-catenin pathway.^125^

Studies have also indicated that Wnt5a plays a role in the immune system and is upregulated in activated macrophages.^101,126^ Palevski et al. found that the expression of Wnt ligands, such as Wnt5a and Wnt11, was significantly increased in macrophages at infarcts during MI in mice, while the β-catenin expression levels remained unchanged during MI. The authors hypothesized that non-canonical Wnt signaling, rather than canonical Wnt signaling, is activated in macrophages at infarct sites.^118^ Following MI in mice, pJNK expression was increased in macrophages at the ischemic edge, the Wnt/PCP pathway was activated, and the Wnt/β-catenin pathway was downregulated, which promoted the transformation of myeloid cells toward a proinflammatory state, thereby aggravating MI.^127^ While Wnt5a is expressed in cardiomyocytes,^128^ in vitro cell culture studies have shown that macrophage-derived Wnt5a activated the non-canonical Wnt/Ca^2+^ signaling pathway via CaMKII and activated NFAT during sepsis.^61^ This signaling pathway induces the expression of pro-inflammatory factors, such as IL-1, IL-6, IL-8, and MIP-1, and enhances inflammatory macrophage activation.^129^

Overall, these findings suggest that targeting the upstream Wnt signaling components or Wnt itself can inhibit the inflammatory response by reversing the Wnt signaling pathway (Fig. 2b). Adeno-associated virus-mediated overexpression of Wnt Inhibitory Factor 1 inhibits the activation of non-canonical Wnt signaling, thereby lowering the expression of IL-1b and IL-6, and exerting an anti-inflammatory effect in heart tissue following acute MI.^127^ To inhibit the expression of RAGE induced by I/R, Park et al. used deoxycholic acid-modified polyethyleneimine as a carrier to introduce RAGE-targeting siRNA into the myocardium. Treatment of rat I/R cardiomyocytes (H9C2 cells) with this PEI-DA/siRAGE complex downregulated the expression of pro-inflammatory cytokines IL-6 and TNF-α, reduced cardiomyocyte apoptosis, and suppressed the infiltration/proliferation of non-cardiomyocytes, exerting anti-apoptotic and anti-inflammatory effects via Wnt/β-catenin activation.^125^

Wntless (Wls) is a conserved multi-channel transmembrane protein that promotes the release of Wnt ligands.^130^ In a mouse model with *Wls*^−/−^ myeloid cells, Wnt signaling was blocked in cardiac macrophages during ischemia and Wnt signaling–mediated macrophage transition toward the M1 phenotype was inhibited, resulting in an accumulation of M2-like macrophages in the MI region. *Wls*–deficient mice have reduced cardiac remodeling and improved cardiac function following MI due to the anti-inflammatory, repair-promoting, and angiogenesis effects of M2 macrophage.^118^ Data on whether canonical or non-canonical Wnt signaling is activated in the cardiac macrophages of ischemic mice remain inconsistent,^119–121,125^ possibly attributed to the different research models used. Indeed, in vivo studies typically indicate that the activation of non-canonical Wnt signaling leads to macrophage polarization,^118,127^ whereas in vitro studies often implicate canonical Wnt signaling activation in macrophage polarization.^119,121,125^ In addition, even when in vivo models are used, the results differ depending on the pathological regions of interest. For example, in the non-infarcted area of the heart, canonical Wnt signaling is dominant, whereas, in the infarct area, non-canonical Wnt signaling is more prominent^125,127^; therefore, the primary signaling pathway that regulates macrophage polarization within the infarct area during I/R may be the non-canonical Wnt signaling pathway.

#### Oxidative stress

Cardiac ischemia leads to Wnt/β-catenin signaling inhibition, oxidative stress elevation, and additional damage to cardiomyocytes (Fig. 2c).

In the cardiomyocytes of ischemic mice, Wnt protein expression is reduced and Wnt/β-catenin signaling is inhibited. As the β-catenin in the cytoplasm is degraded, it cannot enter the nucleus, which blocks the inhibition of the transcription of cytochrome c (cyt c).^78^ The corresponding increase in cyt c aggravates oxidative stress within the cells and mediates an increase in intracellular reactive oxygen species (ROS).^78^ In turn, this excessive production of ROS increases mitochondrial membrane transport channel permeability, mitochondrial membrane potential loss, and cyt c release, ultimately inducing cardiomyocyte apoptosis.^78^ Guo et al. determined that the Wnt/β-catenin pathway was similarly inhibited in ischemic H9C2 cells and also leads to oxidative stress.^131^

These findings suggest that targeting the upstream molecules of the Wnt signaling pathway or directly addressing Wnt can reverse the effects of Wnt signaling and reduce oxidative damage (Fig. 2c). Correspondingly, exogenous Wnt3a administration has been reported to activate the Wnt/β-catenin pathway and inhibit oxidative stress.^78^ Additionally, a peptide derived from tuna backbone protein (APTBP), known for its antioxidant properties, can scavenge ROS.^132^ Under ischemia and I/R injury conditions, APTBP eliminates ROS, restores the activity of Wnt/β-catenin in a dose-dependent manner, protects mitochondria from oxidative stress, and maintains myocardial function.^133^ Alternatively, Guo et al. determined that supplementation of H9C2 cells with induced pluripotent stem cell–derived conditioned medium upregulated Wnt/β-catenin signaling, promoted cardiomyocyte proliferation, and inhibited oxidative stress and cell senescence.^131^

#### ECM remodeling

The Wnt/β-catenin signaling pathway is activated in the myocardium of ischemic mice and promotes ECM remodeling (Fig. 3a).

Four days following experimental MI, β-catenin was upregulated and Wnt/β-catenin signaling was significantly enhanced in mouse subepicardial ECs and mesenchymal cells expressing smooth muscle actin.^122^ Similarly, in mature ECs following MI, the nuclear translocation of β-catenin, upregulation of canonical Wnt signaling response promoters, and activation of canonical Wnt signaling were found to inhibit endothelial markers, induce mesenchymal phenotypes, and upregulate smooth muscle and myofibroblast markers, which are potentially involved in angiogenesis and fibrosis.^122^ Thus, Wnt/β-catenin signaling may be involved in cardiac tissue repair via endothelial–mesenchymal transition (EMT) during MI (Fig. 3a).

#### Angiogenesis

During myocardial I/R, Wnt/β-catenin pathway activation promotes angiogenesis, although some studies have reported that activation of this pathway inhibits angiogenesis. In addition to canonical Wnt signaling, activation of the non-canonical Wnt/PCP pathway is beneficial to angiogenesis (Fig. 3b).

Angiogenesis can repair the injury caused by I/R injury and mitigate cell death. Evidence of intracellular localization of β-catenin indicates that Wnt/β-catenin signaling participates in the proliferation and migration of vascular ECs during neovascularization.^134^ Blankesteijn et al. determined that β-catenin protein was expressed in new blood vessels and original vascular ECs within the infarcted area one-week post MI; additionally, expression of the protein upstream of the Wnt/β-catenin pathway, DVL1, was upregulated in the infarcted rat heart.^134^The authors speculated that the Wnt/β-catenin signaling pathway is activated in the vascular endothelial cells in the infarcted area following ischemia, influencing the proliferation and migration of vascular ECs during neovascularization.^134^ In AMI mice, the canonical Wnt pathway was activated at the infarct area, β-catenin accumulated in the cardiac vascular cells, the capillary density of the ischemic scar was reduced, and cardiac function damage was aggravated.^135,136^

Wang et al. established that after the left anterior descending coronary artery (LAD) ligation, Wnt11 expression in rat myocardial tissue significantly decreased in a time-dependent manner, while infarct size increased; following reperfusion, capillary-like tube formation and human umbilical vein endothelial cells angiogenesis were also observed. The authors postulated that the activation of atypical Wnt11/PCP pathway increases angiogenesis and improves cardiac function.^50^

These findings suggest that targeting the Wnt signaling pathway can promote vascular regeneration following cardiac I/R injury (Fig. 3b). The GSK-3β allosteric inhibitor NP12, which stabilizes β-catenin and activates the Wnt signaling pathway, promotes angiogenesis, and improves cardiac function during MI.^136^ In primary mouse aortic ECs, glucocorticoid receptor (GR) deficiency promotes angiogenesis. Under these conditions, GR deficiency activates Wnt/β-catenin signaling by facilitating the binding of LRP5/6 to Wnt3a, leading to the accumulation of β-catenin in the nucleus and resulting in the upregulation of the angiogenic regulator cyclin D1.^137^ Alternatively, the addition of Wnt11 activated the atypical Wnt11/PCP pathway to upregulated angiogenesis.^50^

#### Cell hypertrophy

Cardiomyocytes exit the cell cycle and begin terminal differentiation shortly after birth.^138^ Therefore, in the adult heart, the increase in cardiomyocyte size, rather than number, induces hypertrophy. This hypertrophy helps to reduce wall pressure and maintain cardiac function and efficiency in response to increased workload.^139^ However, pathological hypertrophy can occur under adverse stimulation conditions, such as myocardial ischemia, and lead to maladaptive cardiac remodeling and heart failure.^140^ In myocardial I/R, Wnt/β-catenin signaling is inhibited and cell hypertrophy is promoted, by the Wnt/PCP signaling pathway^61^ (Fig. 3c).

Dpr1 is necessary for Wnt5a signaling, which induces cardiomyocyte hypertrophy and activates Wnt/PCP signaling in cardiomyocytes.^141^ Localization of the Wnt/PCP transmembrane receptor Van-Gogh-like-2 (Vangl2) is predominantly within the membrane and cytoplasm; however, in cells without Dpr1, Vangl2 has been shown to significantly accumulate within vesicles in the perinuclear region.^141^ During ischemic injury, the Wnt5a /PCP pathway is activated by the ROR2/Vangl2/JNK axis, and Wnt/β-catenin signaling is inhibited, thus promoting post-ischemic myocardial hypertrophy.^141^ Following I/R injury, the cell hypertrophy of AC16 human left ventricular cardiomyocytes is exacerbated, Wnt/β-catenin signaling is inhibited, and the expression of hypertrophy markers, including myosin heavy chain 7, brain natriuretic peptide, and endothelin 1, is significantly increased.^142^

Overall, therapy targeting the Wnt/β-catenin signaling pathway may potentially mitigate cellular hypertrophy and reduce myocardial remodeling and heart failure. For instance, treatment with CHIR99021, a GSK-3β inhibitor, following MI activates Wnt/β-catenin signaling, which, in conjunction with the Yes-associated protein (YAP) pathway, can alleviate cardiomyocyte hypertrophy.^142^

#### Fibrosis

In the normal heart, fibroblasts remain quiescent and are predominantly involved in the daily maintenance of the ECM. These cells are activated and significantly expand following ischemic myocardial injury, thereby initiating excessive ECM remodeling and fibrosis. Interestingly, the Wnt/β-catenin signaling pathway is activated in the hearts of ischemic mice and promotes fibrosis (Fig. 3c).

Zhao et al. established a rat ischemia model via ligation of LAD ligation model. The authors established that the expression levels of Wnt1, β-catenin, and phosphorylated GSK-3β were significantly higher in this ischemic model than those in the corresponding control group; further, ischemic rats exhibited left ventricular dysfunction, pathological heart failure, and signs of cardiac remodeling. The authors attributed these changes to the corresponding activation of Wnt/β-catenin signaling after MI.^143^ Similarly, Qian et al. found that the expression levels of β-catenin and cardiac fibrosis markers were significantly increased in hypoxic cardiac fibroblasts cultured in vitro and ischemia mouse models, alongside observations of aggravated myocardial fibrosis, which was attributed to the activation of Wnt/β-catenin signaling.^144^ In addition, *miR‐145* expression was lower in MI rats and hypoxic CFs, which was accompanied by cardiac dysfunction and excessive fibrosis in vivo, and activated CFs in vitro.^145^ Cui et al. determined that *miR-145* could directly target sex-determining region Y box 9 (SOX9) in fibroblasts and reduce cardiac fibrosis by downregulating the canonical Wnt signaling pathway in an ischemic rat model.^145^ Further, Matsushima et al. discovered that the left ventricular wall of the rat heart decreased on thickness during ischemia, while the left ventricular cavity increased significantly; these effects were similarly accompanied by an increase in inactivated GSK-3β expression and β-catenin activity. This activation of β-catenin stimulated the proliferation of collagen-producing cells at the ischemic edge and, ultimately, promoted fibrosis.^146^ Following acute ischemic heart injury, Wnt1 expression is upregulated, which induces the proliferation of cardiac fibroblasts, increases the expression of pro-fibrotic genes by activating Wnt1/β-catenin signaling, and promotes cardiac repair.^147^ In contrast, inhibition of Wnt1/β-catenin signaling in cardiac fibroblasts can impair cardiac function and ventricular dilatation.^147^

These findings ultimately suggest that targeting factors upstream of Wnt and its corresponding signaling pathways can reduce myocardial fibrosis (Fig. 3d). Zhang et al. established AMI mouse and cell culture models within an oxygen-glucose deprivation environment. Proline/arginine-rich terminal leucine repeat protein was found to increase myocardial infarct size following ischemia, both in vivo and in vitro, by activating the downstream Wnt/β-catenin signaling pathway and promoting myocardial fibrosis and ventricular remodeling.^148^ Knockouts of S100A4, a calcium-binding protein observed in mouse cardiac fibroblasts and cardiomyocytes, showed a significant reduction in β-catenin levels and cardiac fibrosis.^144^ Matsushima et al. demonstrated that *sFRP-4* expression was upregulated in the ischemic border region of LAD-ligated rat hearts.^146^ Following ischemia and reperfusion injury, administration of sFRP-4 protein to rat intracardiac muscle improved cardiac function. Histological and immunohistochemical staining of cardiac sections from the untreated (without sFRP-4) rats indicated that following I/R injury, the left ventricular wall thickness decreased, left ventricular cavity size significantly increased, deactivated GSK-3β levels increased, and β-catenin activity was upregulated. Overall, this stimulated the proliferation of collagen-producing cells at the ischemic border region and promoted fibrosis. In contrast, the size of the left ventricular cavity in sFRP-4-treated rat hearts did not increase in size; further, sFRP-4 treatment at the early stages of ischemia could inhibit cell proliferation and reduce cardiac fibrosis by inhibiting Wnt/β-catenin signaling activation.^146^

### Wnt pathways during cerebral I/R injury

The timely identification and intervention of ischemic stroke are of utmost importance. The current recommended therapeutic strategy for ischemic stroke involves thrombolysis, via recombinant tissue plasminogen activator (rtPA) injection within 4.5 h post onset,^149–152^ alongside mechanical thrombectomy within 24 h post onset.^150,151,153^ Extending the treatment time window has been a focus of previous studies, and conflicting results on the clinical effects of bridging therapy (intravenous thrombolysis with rtPA before mechanical thrombectomy) have been reported^154–157^; leading to ongoing controversy.^158^

Cerebral I/R injury usually occurs during reperfusion therapy for cerebrovascular diseases such as ischemic stroke. The Wnt signaling pathway controls the proliferation, differentiation, and migration of neurons, the development of neural crests, the growth of axons and dendrites, and the maintenance of angiogenesis and the BBB within the mammalian embryonic and postnatal brain.^159–166^ Nonetheless, this Wnt-mediated regulation can continue into adulthood. During cerebral I/R injury, the Wnt signaling pathway transitions from an activated state to an inhibited state as ischemia time increases, and is regulated by many processes such as autophagy.^10,29,149,167,168^ The inhibitory Wnt signaling pathway is primarily associated with apoptosis, ferroptosis, neurogenesis, angiogenesis, BBB damage, inflammatory responses, and oxidative stress. Abnormal activation or inhibition of the Wnt signaling pathway has been observed in different cell types within the nervous system, including neurons, microglia, astrocytes, oligodendrocytes, and ECs. Therefore, targeting this pathway holds potential for mitigating cerebral ischemia and subsequent reperfusion injury.

#### Apoptosis

**Fig. 4:**
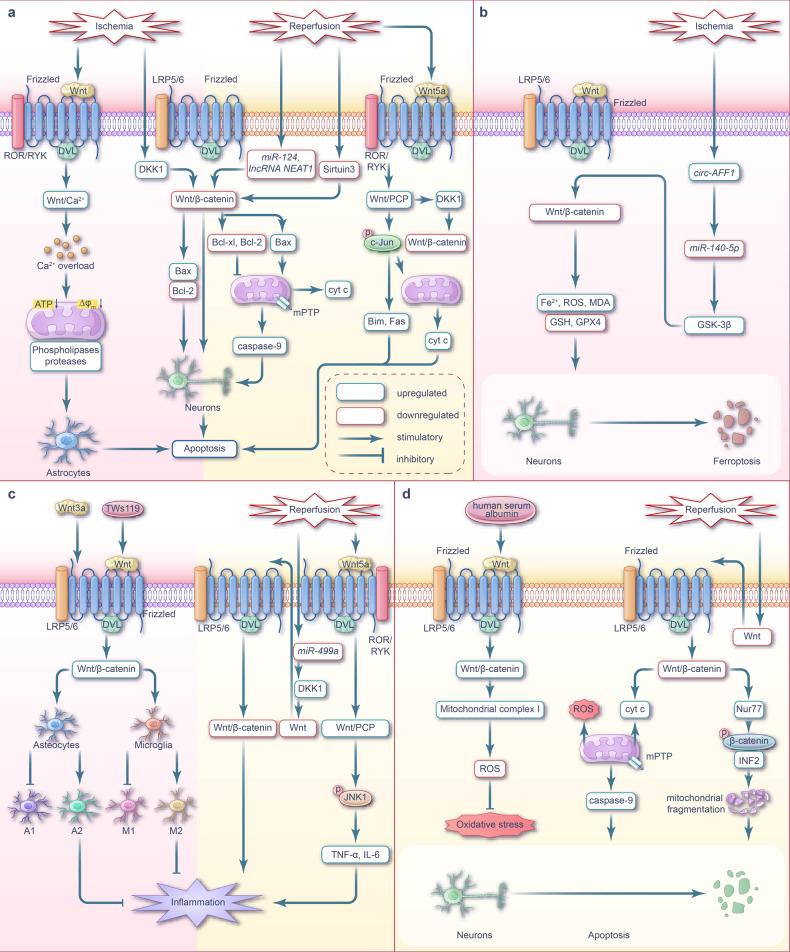
Wnt signaling pathway and targeted therapy for apoptosis, ferroptosis, inflammation, and oxidative stress during cerebral I/R injury. **a** Wnt signaling-mediated apoptosis during cerebral I/R injury. During cerebral ischemic phase, Wnt/Ca^2+^ signaling is activated, leading to intracellular calcium overload and and subsequent astrocyte apoptosis. The upregulation of DKK1 inhibits Wnt/β-catenin pathway, leading to neuronal apoptosis. During cerebral I/R phase, Wnt5a-mediated Wnt/PCP signaling is activated, promoting c-Jun phosphorylation, inducing cyt c release from mitochondria, inhibiting Wnt/β-catenin signaling, and ultimately leads to neuronal apoptosis. Downregulation of Sirtuin3, *miR-124* and lncRNA *NEAT1* also inhibit Wnt/β-catenin signaling. **b** Wnt signaling-mediated ferroptosis during cerebral I/R injury. During cerebral ischemia phase, *circ-AFF1* is highly expressed and directly targets *miR-140-5p* to upregulate GSK-3β. The highly expressed GSK-3β inhibits Wnt/β-catenin signaling. The inhibition of Wnt/β-catenin signaling leads to excessive accumulation of Fe^2+^, ROS and MDA, and suppression of GSH and GPX4 expression, thereby aggravating neuronal ferroptosis. **c** Wnt signaling-mediated inflammation during cerebral I/R injury. During cerebral ischemic phase, Wnt/β-catenin signaling is activated, which promotes the polarization of reactive microglia to M2 phenotype, increases the number of A2 type of astrocytes, and reduces the number of A1 type of astrocytes, thereby playing a protective effect and reducing the inflammatory response caused by cerebral ischemia. During cerebral I/R phase, downregulation of *miR-499a* leads to inhibition of Wnt/β-catenin signaling, thereby aggravating the inflammatory response. Wnt5a-mediated Wnt/PCP signaling is activated during cerebral I/R, leading to upregulation of the pro-inflammatory cytokines, thus aggravates the inflammatory response during cerebral I/R. **d** Wnt signaling-mediated oxidative stress during cerebral I/R injury. During cerebral I/R phase, Wnt/β-catenin signaling is inhibited which lead to neuronal apoptosis *via* mitochondria dysfunction. The expression of Nur77 is stimulated in the oxidative stress environment during cerebral I/R, which leads to mitochondrial fragmentation by promoting β-catenin phosphorylation and INF2 expression. Intravenous injection of human serum albumin activates Wnt/β-catenin signaling, thereby increasing mitochondrial complex I activity, reducing ROS generation, suppressing oxidative stress, and playing a therapeutic role during cerebral I/R. Note: The pink background represents the ischemic phase, while the cream background represents the reperfusion phase. LRP low-density lipoprotein receptor-related protein, ROR recombinant receptor tyrosine kinase like orphan receptor, RYK receptor tyrosine kinase, ATP adenosine triphosphate, Δφ membrane potential, DKK1 Dickkopf-1, cyt c cytochrome-c, GSH glutathione, GPX4 glutathione peroxidase 4, GSK-3β glucogen synthase kinase 3β, JNK1 c-Jun amino-terminal kinase1, TNF-α tumor necrosis factor-α, TWS119 a GSK-3β inhibitor that activates Wnt/β-catenin signaling, Nur77 nuclear hormone receptor NUR/77, INF2 inverted formin 2, mPTP mitochondrial permeability transition pore

Cerebral I/R injury inhibits Wnt/β-catenin signaling, which contributes to neuronal apoptosis.^169,170^ However, compensatory activation of the Wnt/β-catenin signaling pathway can occur during early ischemia to counteract apoptosis.^171^ Previous studies have shown that during cerebral I/R injury, Wnt/PCP signaling is initially activated before being inhibited, which can antagonize Wnt/β-catenin signaling and accelerate apoptosis. This early activation of Wnt/PCP signaling may be a significant factor contributing to the inhibition of Wnt/β-catenin signaling.^29^ Changes in Wnt/Ca^2+^ signaling during cerebral I/R injury remain unclear; however, the intracellular calcium overload induced by its activation is a crucial mechanism of apoptosis^172^ (Fig. 4a).

In the hours to days following acute cerebral ischemia or traumatic brain injury, neuronal and glial cell apoptosis is initiated, predominantly in the ischemic penumbra; while rapid cell necrosis occurs in this ischemic core.^173^ DKK1, a negative regulator of Wnt/β-catenin signaling, is elevated in the plasma of patients with ischemic stroke and in the neurons of cerebral ischemia animal models.^174–176^ Specifically, DKK1 has been reported to bind to LRP5/6, activate GSK-3β, inhibit Wnt/β-catenin signaling, and promote neuronal apoptosis by increasing the expression of the pro-apoptotic protein Bax and reducing the expression of the anti-apoptotic protein Bcl-2.^177^ During cerebral I/R, Wnt/β-catenin signaling is inhibited by a decrease in Sirtuin3 levels^169^ and downregulation of *miR-124*^178^ and lncRNA *NEAT1*,^179^ ultimately leading to neuronal apoptosis.

Previous studies using mouse and rat cerebral I/R and cellular H/R models have reported that the downregulation or inactivation of Wnt/β-catenin signaling can induce the expression of the pro-apoptotic protein Bax and downregulate the anti-apoptotic protein Bcl-2, promoting neuronal apoptosis.^169,170,180,181^ Specifically, Bax promotes the release of cyt c from the mitochondria, whereas Bcl-xL and Bcl-2 inhibit this process.^182^ During cerebral I/R injury, the inhibition of Wnt/β-catenin signaling leads to mitochondrial damage, including excessive mitochondrial fission,^169^ cyt c release, caspase 9 activation,^181^ and subsequent caspase three activation, ultimately resulting in cell apoptosis.^182^ Li et al. demonstrated that in vitro neuronal oxygen-glucose deprivation/reoxygenation (OGD/R) treatment activated the Wnt/β-catenin signaling pathway and resulted in apoptosis inhibition and improved neuronal survival. Correspondingly, the authors suggested that this may be an early adaptive response to cerebral I/R injury.^171^

The non-canonical Wnt/PCP and Wnt/Ca^2+^ signaling pathways also play key roles in apoptosis following cerebral I/R injury. JNK3 is a major JNK subtype activated in cerebral ischemia, while β-arrestin2 is a scaffold protein involved in the regulation of JNK3 signaling.^183^ Wei et al. showed that Wnt5a expression increased after cerebral I/R in rats but decreased 24 h later. Additionally, the interaction between Dvl-1, β-arrestin2, and JNK3 was enhanced 3 h post-reperfusion. Wnt5a promotes the assembly of the Dvl-1–arrestin2–JNK3 module, thereby activating JNK3 and promoting c-Jun phosphorylation.^29^ Whether JNK-related signals promote or inhibit apoptosis depends on various conditions. JNK3 activation promotes the release of mitochondrial cyt c and induces the transcription of pro-apoptotic proteins, such as Bim and Fas, and their corresponding receptor genes, resulting in neuronal apoptosis.^184^ Zhang et al. demonstrated that JNK/c-Jun pathway activation can induce the expression of DKK1, thus inhibiting the canonical Wnt pathway.^185^ The increased expression of Wnt5a not only activates the Wnt/PCP pathway, but also upregulates DKK1—which has an antagonistic effect on canonical Wnt signaling—jointly mediating the occurrence of apoptosis and aggravating cerebral I/R injury.^29^ Niu et al. reported that activation of Wnt/Ca^2+^ signaling during brain injury resulted in calcium overload and cell death in hippocampal astrocytes.^172^ Cerebral ischemia increases H^+^ levels in brain cells, thereby opening acid-sensitive ion channels. The acid-sensing ion channel is a dual-ligand gated channel using Ca^2+^ and H^+^; therefore, its activation leads to Ca^2+^ overload in brain cells during cerebral ischemia. Abnormally high levels of Ca^2+^ eliminate mitochondrial oxidative phosphorylation, reduce mitochondrial membrane potential and adenosine triphosphate content, activate phospholipases and proteases, and cause irreversible damage to brain cells.^186^

Overall, targeting the Wnt signaling pathway can potentially mitigate apoptosis caused by cerebral I/R injury (Fig. 4a). Isoflurane, an inhalational anesthetic, exhibits neuroprotective effects in ischemic and hypoxic brain injury.^187^ In a rat cerebral I/R injury model, isoflurane postconditioning (inhalation of 1.5% isoflurane for 60 min following reperfusion) could activate the Wnt/β-catenin signaling pathway and inhibit neuronal apoptosis.^170^ In addition, similar effects have been reported in animal and cell models of cerebral I/R that were treated with ginkgolide B derivative XQ-1H by gavage^180^ and oxymatrine via intraperitoneal injection.^188^

#### Ferroptosis

Ferroptosis is a form of cell death driven by oxidative stress and iron overload. Cerebral I/R injury-initiated ferroptosis is regulated by Wnt/β-catenin signaling. Therefore, Wnt/β-catenin signaling is a promising target for cerebral I/R injury treatment (Fig. 4b).

Yan et al. demonstrated that neuronal ferroptosis occurred during the reperfusion of cerebral I/R. Ferroptosis is associated with an increase in brain-derived, rather than blood-derived, thrombin and the subsequent release of arachidonic acid.^189^ Acyl-CoA synthetase long-chain family member 4 (ACSL4) can catalyze the formation of CoA from arachidonic acid, resulting in an accumulation of lipid peroxides and, ultimately, triggering ferroptosis.^190^ Timely downregulation of ACSL4 during cerebral I/R exhibits a protective role via the initiation or inhibition of thrombin elevation, which can reduce subsequent neuronal ferroptosis.^189^ Additionally, neuronal ferroptosis causes iron overload^191–193^ and decreases GPX4 expression^194^ in the brain tissue during cerebral ischemia. The application of iron chelators^195,196^ or drugs that improve iron metabolism, such as lycopene,^197^ to reduce brain iron levels can alleviate cerebral I/R injury. Notably, selenium can promote mitochondrial fusion by inducing Mfn1 expression, thereby improving the oxidative stress and ferroptosis caused by brain I/R in mice.^194^

In cerebral hemorrhage injury, downregulation of Wnt/β-catenin signaling contributes to neuronal ferroptosis.^198^ Nuclear erythroid 2-related factor 2 (Nrf2) is a transcription factor associated with antioxidant stress. GSK-3β reduces the expression of GPX 4 and induces ROS generation,^199^ which initiates a cytotoxic response caused by oxidative stress during dopaminergic neuronal death progression via the inhibition of Nrf2 signaling.^200^

Targeting GSK-3β to activate the Wnt/β-catenin signaling pathway is a potential therapeutic strategy to curb ferroptosis in cerebral I/R injury (Fig. 4b). For example, the knockdown of the *circAFF1* gene can upregulate *miR-140-5p*, thereby reducing GSK-3β expression, which in turn activates the Wnt/β-catenin signaling pathway.^198^ The activated Wnt/β-catenin signaling pathway reduces the accumulation of Fe^2+^, ROS and malondialdehyde, and induces the expression of glutathione and GPX4, thus inhibits neuronal ferroptosis.^198^ Wang et al. demonstrated that direct knockout of GSK-3β could downregulate the expression of the divalent metal transporter 1, ferritin heavy chain, and ferritin heavy chain polypeptide 1 genes, reduce the number of intracellular free iron, and initiate anti-ferroptosis mechanisms. The authors attributed these findings to the GSK-3β knockout preventing the activation of downstream Wnt/β-catenin signaling.^201^

#### Inflammatory response

The Wnt/β-catenin signaling pathway is inhibited during cerebral I/R, which exacerbates the pro-inflammatory response. The non-canonical Wnt/PCP signaling pathway is also activated following cerebral I/R and may also aggravate inflammation via unclear mechanisms (Fig. 4c).

The inflammatory response following cerebral ischemia exhibits a dual role. On one hand, the release of inflammatory mediators initiates acute BBB disruption and neuronal damage, while on the other hand, inflammation plays a vital role in the repair process during ischemia.^202^ The rapid expression of multiple inflammatory factors in patients with ischemic stroke has been determined to aggravate BBB injury. This damaged BBB is the site of neutrophil and monocyte infiltration and matrix metalloproteinase 9 (MMP9) release.^203^ Microglia, the innate immune effector cells of the central nervous system, are activated during ischemic stroke.^202,204,205^ In the early stage of cerebral ischemia, microglia undergo a phenotype switch from the anti-inflammatory M2 phenotype to the pro-inflammatory M1 phenotype.^206,207^ The Wnt signaling pathway is involved in the toll-like receptor (TLR)-mediated central nervous system immune response.^208^

Wnt protein is expressed in microglia. For instance, Wnt5a expression in microglia activates the non-canonical Wnt signaling pathway, which initiates an immune response against nerve injury by increasing the genetic expression of TNF-α, IL-6, and IL-1β.^209^ Microglia in different activation states secrete distinct Wnt proteins; for example, M1 phenotype microglia secrete Wnt5a, whereas M2 phenotype microglia secrete Wnt7a.^210^ The Wnt signaling pathway has been implicated in the regulation of the neuro-inflammation caused by cerebral I/R injury. In experimental multiple sclerosis, activation of Wnt/β-catenin signaling reduced neutrophil and monocyte infiltration and limited the progression of neuro-inflammation.^203^ Wnt/β-catenin signaling is inhibited in ischemic stroke patients and corresponding mouse models, which contributes to the release of inflammatory factors TNF-α, IL-1, IL-6, and IL-8, and aggravates the inflammatory response.^211^ During cerebral I/R, *miR-499a* downregulation mitigates the inhibition of downstream target DKK1, which further suppresses Wnt/β-catenin signaling and exacerbates the inflammatory response.^212^ In H/R-treated neurons, the Wnt5a-mediated Wnt/PCP signaling pathway is activated and JNK1 is phosphorylated; consequently, an increase in the expression of pro-inflammatory cytokines TNF-α and IL-6 is observed, which amplifies the inflammatory responses.^213^ Alternatively, Wnt/β-catenin signaling activation inhibits the inflammatory response during I/R; however, the mechanism by which the non-canonical Wnt/PCP signaling pathway regulates neuro-inflammation during cerebral I/R injury remains unclear; therefore, further research is warranted. One possibility is that Wnt5a drives the non-canonical Wnt signaling pathway and induces inflammation by promoting microglial polarization toward the M1 phenotype.^209,210^

Targeting the Wnt signaling pathway or related proteins is a potential strategy to reduce the damage caused by the inflammatory response in cerebral I/R injury (Fig. 4c). TWS119 is a GSK-3β inhibitor that activates Wnt/β-catenin signaling. On days 14 and 21 following experimental ischemic stroke, TWS119 treatment promoted microglial polarization by activating Wnt/β-catenin signaling, which ultimately improved the local inflammatory microenvironment during the chronic phase of ischemic stroke. These observations are accompanied by angiogenesis surrounding the infarct area.^214^

Alternatively, intranasal administration of Wnt3a reduced the volume of cerebral infarction and the number of apoptotic cells 72 h post-transient middle cerebral artery occlusion (MCAO) in mouse models. Additionally, this treatment promoted the polarization of reactive microglia toward the M2 phenotype; increased the number of A2 phenotype astrocytes with neuroprotective effects; reduced the number of neurotoxic A1 phenotype astrocytes; initiated the anti-inflammatory and neuroprotective effects of microglia and astrocytes; reduced the neuroinflammation following cerebral ischemia, which may be attributed to the Wnt3a-mediated activation of the Wnt/β-catenin signaling pathway.^215^ Finally, treatment with curcumin has shown efficacy in reversing the inflammatory response caused by Wnt/PCP signaling activation in neuronal cells subjected to H/R.^216^

#### Oxidative stress

Oxidative stress plays a crucial role in brain injury following cerebral I/R injury, and inhibition of the Wnt/β-catenin signaling pathway is a key factor in the pathogenesis (Fig. 4d).

One study demonstrated that rat cerebral I/R injury inhibited Wnt/β-catenin signaling; this signal inhibition decreased the activity of mitochondrial complex I and caused an oxidative stress state via excessive ROS generation, which contributed to brain injury.^217^ During reperfusion, the rate of mitochondrial ROS production increases, resulting in the opening of mitochondrial permeability transition pores and subsequent cell death.^216,218^ Further, brain I/R reduces antioxidant levels and leads to excessive production of mitochondrial ROS, which damages the mitochondrial membrane, triggers the release of cyt c and expression of caspase 9, and ultimately induces neuronal apoptosis.^181^

The inverted formin two protein is required for excessive mitochondrial fission in mammalian cells.^181,219^ In the oxidative stress environment of cerebral I/R, the expression of the nuclear hormone receptor Nur77 is stimulated, promoting β-catenin phosphorylation and a subsequent increase in inverted formin two expression, which leads to mitochondrial fragmentation,^181^ an overactivation of mitochondrial fission and inhibition of fusion. This phenomenon mediates mitochondrial and neuronal cell damage during cerebral I/R, and consequently aggravates brain injury.^181^ Intravenous injection of human serum albumin can activate the Wnt/β-catenin signaling pathway and reduce early oxidative stress injury following cerebral I/R injury of rats.^217^

#### Neurogenesis

**Fig. 5:**
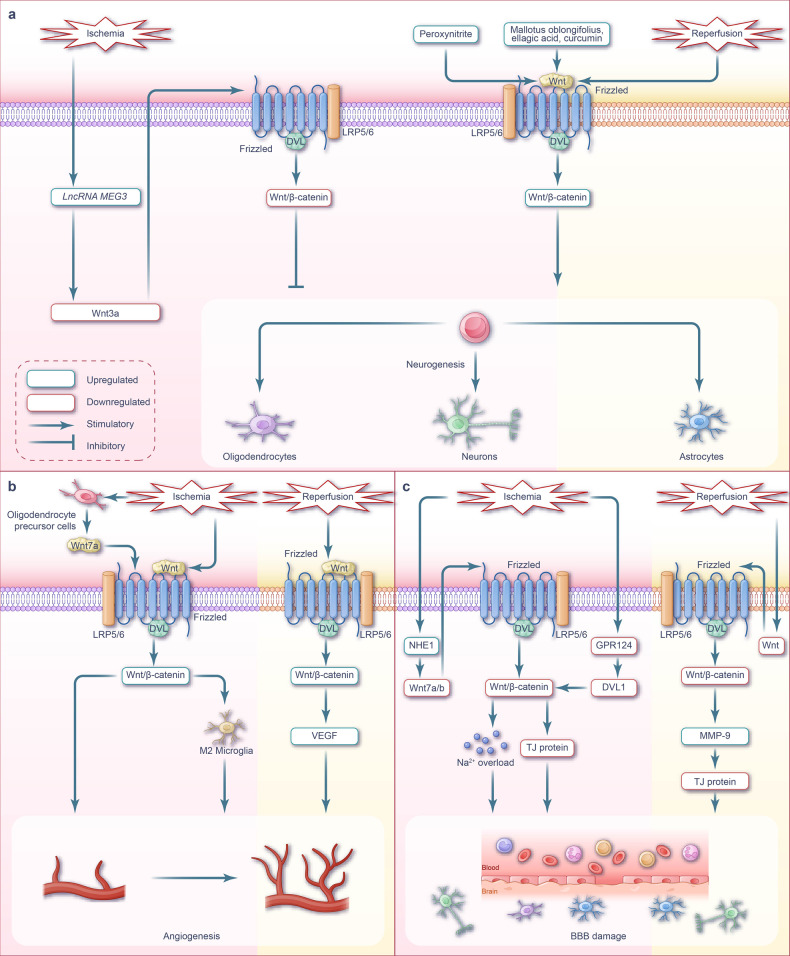
Wnt signaling pathway and targeted therapy for neurogenesis, angiogenesis, and BBB during cerebral I/R injury. **a** Wnt signaling-mediated neurogenesis during cerebral I/R injury. During cerebral ischemia phase, there is an elevation in the synthesis of lncRNA *MEG* and peroxynitrite. The highly expressed lncRNA *MEG* hampers the process of the Wnt/β-catenin signaling pathway. In contrast, increased levels of peroxynitrite activate the Wnt/β-catenin signaling pathway. In the subsequent phase of cerebral I/R phase, the activation of the Wnt/β-catenin signaling pathway plays a crucial role in promoting neurogenesis. Mallotus oblongifolius, ellagic acid, and curcumin enhance the activation of the Wnt/β-catenin signaling, leading to the promotion of neurogenesis and the exertion of therapeutic effects on cerebral ischemia or I/R injury. **b** Wnt signaling-mediated angiogenesis during cerebral I/R injury. Following cerebral ischemia, oligodendrocyte precursor cells within the brain secrete Wnt7a, which triggers the activation of Wnt/β-catenin signaling specifically in endothelial cells. This activation, in turn, facilitates the process of angiogenesis. Activation of the Wnt/β-catenin signaling pathway induces the conversion of microglia into the M2 phenotype, thereby facilitating angiogenesis following an ischemic stroke. Following cerebral I/R, the activation of the Wnt/β-catenin signaling pathway upregulates the expression of VEGF and VEGF receptors. This activation promotes angiogenesis and stimulates the proliferation and sprouting of vascular endothelial cells. **c** Wnt signaling-mediated the BBB during cerebral I/R injury. The mutation or deletion of GPR124 leads to a reduction in the recruitment of DVL1 to the cell membrane. Consequently, this weakens the transduction of Wnt/β-catenin signaling, resulting in the downregulation of TJ protein expression between microvascular endothelial cells. As a consequence, it exacerbates the damage to the BBB following cerebral ischemia. Moreover, during the cerebral ischemic phase, there is an upregulation of NHE1, which inhibits Wnt/β-catenin signaling and disrupts astrocyte function. This disruption is necessary to maintain the integrity of the BBB. In the context of cerebral ischemia, NHE1 undergoes upregulation, which subsequently inhibits Wnt/β-catenin signaling and disrupts the function of astrocytes. This disruption ultimately results in impaired BBB integrity. During the cerebral I/R phase, there is inhibition of Wnt/β-catenin signaling, leading to an increase in the expression of MMP-9. This elevated MMP-9 expression subsequently degrades the TJ proteins between brain endothelial cells, disrupting the integrity of the BBB. Note: The pink background represents the ischemic phase, while the cream background represents the reperfusion phase. LRP low-density lipoprotein receptor-related protein, VEGF vascular endothelial growth factor, BBB blood-brain barrier, GPR124 G protein-coupled receptor 124, TJ protein tight junction protein, MMP-9 matrix metalloproteinase-9, NHE1 the protein encoded by the *Nhe1* gene

The activation of Wnt/β-catenin signaling promotes neurogenesis during the early stage of cerebral ischemia and I/R injury. However, the inhibition of Wnt/β-catenin signaling pathway hinders the protective effect. Therefore, targeting the Wnt/β-catenin signaling pathway is a potential treatment for restoring neurogenesis following cerebral I/R injury (Fig. 5a).

The Wnt signaling pathway is a key regulatory pathway of neurogenesis. In the forebrain tissue of embryonic mice on embryonic day 14.5, it promotes the self-renewal of neural stem cells, inhibits neural stem cell differentiation, and retains the pluripotency of neural stem cells, which allows the cells to differentiate into neurons, astrocytes, and oligodendrocytes.^159^ The canonical Wnt signaling pathway also promotes the differentiation of mice cortical neurons on embryonic day 10.5.^160^ Additionally, the non-canonical Wnt/PCP signaling pathway, activated by tyrosine kinase receptors, regulates the production of different subtypes of cortical interneurons located in the medial ganglion eminence during embryonic development.^161^ Thus, the Wnt signaling pathway is essential for embryonic neural stem cell proliferation and neuronal differentiation. In the adult brain, neurogenesis occurs in the subventricular zone (SVZ) of the lateral ventricle and subgranular zone of the dentate gyrus in the hippocampus.^220,221^ Wnt/β-catenin signaling is activated in these regions^222,223^ and is involved in neurogenesis. Jin et al. observed a significant increase in proliferation-related Ki-67 antigen-positive cells in the ischemic penumbra in the autopsy brain tissue of adult patients with ischemic stroke, alongside the expression of neuronal lineage doublecortin, tOAD/Ulip/CRMP family protein 4, and βIII tubulin.^224^ Another study demonstrated that cell proliferation was active in the SVZ region in elderly patients who died of ischemic stroke;^225^ which indicated that cerebral ischemic injury-induced adult neurogenesis is possible. Notably, the Wnt/β-catenin signaling pathway is widely activated during cerebral I/R injury, to possibly promote neurogenesis via the upregulation of various downstream molecules. Neurogenesis alleviates brain injury by combating apoptosis.

Cerebral hypoxic-ischemic injury increases peroxynitrite production and promotes neural stem cell proliferation and neuronal differentiation. This has been partially attributed to peroxynitrite activating the Wnt/β-catenin signaling pathway; however, when peroxynitrite production reaches a specific threshold, it exerts a cytotoxic effect.^226^ Instead, artificially activating the Wnt/β-catenin signaling pathway can promote the expression of the downstream targets, cyclin D1, Ngn2, Pax6, and NeuroD1, thereby promoting neurogenesis without the cytotoxic effects of peroxynitrite. Cyclin D1^227^ and Pax6^228^ promote neural stem cell proliferation, Neuro D1 promotes adult neurogenesis and maintains neuronal survival,^229,230^ and Ngn2 promotes neurogenesis.^231^ Additionally, Wnt/β-catenin signaling activates BDNF secretion from glial cells and protects adjacent neurons.^232^ Overall, this upregulation of BDNF by Wnt/β-catenin signaling contributes to nerve repair during an ischemic stroke by promoting neurogenesis and neuronal survival.^233^

Several substances, such as *Mallotus oblongifolius*,^234^ ellagic acid,^235^ and curcumin,^236^ have been shown to upregulate Wnt/β-catenin signaling during cerebral ischemia and cerebral I/R injury, promoting neurogenesis by activating the downstream targets cyclin D1, Ngn2, Pax6, and NeuroD1. Alternatively, long non-coding RNA *MEG3* (lncRNA *MEG3*) expression increases during cerebral ischemia, and the downregulation of lncRNA *MEG3* expression activates Wnt/β-catenin signaling and promotes neurogenesis.^237^

#### Angiogenesis

During cerebral I/R injury, artificial activation of the Wnt/β-catenin signaling pathway can promote angiogenesis (Fig. 5b). Specifically, in the mammalian embryonic forebrain, angiogenesis relies on the activation of Wnt signaling in vascular ECs, driven by Wnt7a and Wnt7b. Angiogenesis in the hindbrain is initiated by the binding of the Norrin ligand to the Frizzled 4 receptor, activating the β-catenin signaling pathway and promoting angiogenesis.^238,239^ Zhang et al. demonstrated that activation of Wnt/β-catenin signaling during cerebral I/R could promote the expression of vascular endothelial growth factor (VEGF),^170^ which plays a dual role in cerebral ischemia by transiently destroying the BBB and promoting angiogenesis.^240^ Wnt/β-catenin signaling promotes the proliferation and sprouting of vascular ECs and increases the expression of VEGF receptors, thereby promoting angiogenesis within the central nervous system.^241^ Wnt/β-catenin signaling induces the polarization of reactive microglia toward the M2 phenotype.^242^ M2 microglia secretes exosomes containing *miRNA-26a*, which targets ECs and promote angiogenesis during ischemic stroke.^242^ Angiogenesis in the penumbra of early cerebral ischemia in patients with ischemic stroke is significantly increased and correlated with patient survival.^243^ Wnt/β-catenin signaling activation during cerebral I/R is a potential strategy for enhancing angiogenesis. During cerebral ischemia, transplanted oligodendrocyte precursor cells secrete Wnt7a in a paracrine manner; Wnt7a can activate EC Wnt/β-catenin signaling and promote angiogenesis and neurological recovery.^244^ Alternatively, isoflurane postconditioning (inhalation of 1.5% isoflurane for 60 minutes after reperfusion) can activate the Wnt/β-catenin signaling pathway and promote the expression of target protein VEGF,^170^ which may promote angiogenesis.

#### BBB

Inhibition of Wnt/β-catenin signaling exacerbates BBB damage during cerebral I/R (Fig. 5c). Wnt/β-catenin signaling is activated in cerebral vessel ECs from the embryonic stage to postpartum BBB formation; however, its transduction is reduced as the BBB matures.^165^ Wnt/β-catenin signaling is silent in capillary ECs within circumventricular organs owing to the maintenance of highly permeable capillaries in this region.^166^ Vascular edema during ischemic stroke is a primary contributor of BBB breakdown. BBB dysfunction is characterized by increased permeability that allows blood-derived fluids and chemicals to enter the brain parenchyma, ultimately resulting in brain edema.^245^ Ta et al. demonstrated that two single-nucleotide polymorphisms in Wnt7a and three single-nucleotide polymorphisms in the adhesion G protein-coupled receptor GPR124 were associated with an increased risk of hemorrhagic transformation following rtPA thrombolysis in patients with acute ischemic stroke. Specifically, a GPR124 c.3587G>A mutation reduced GPR124-mediated recruitment of sufficient DVL1 from the cytoplasm to the cell membrane, thereby reducing the interaction between DVL1 and Wnt receptors and weakening the Wnt signaling pathway.^246^ Additionally, *GPR124* gene deletion within ECs worsens BBB damage during cerebral ischemia^247^ by downregulating Wnt/β-catenin signaling; nonetheless, this effect gradually diminishes in transient MCAO mice through pericyte shedding and reperfusion from day 3–5.

Correspondingly, a decrease in TJ protein expression disrupts BBB integrity and increases the risk of hemorrhagic transformation following reperfusion.^247^ In animal models, Wnt/β-catenin signaling is reportedly attenuated during cerebral I/R, leading to increased BBB permeability and damage due to the downregulation of TJ protein in microvascular ECs.^248,249^ Capillary ECs are the main components of the BBB, with TJs being observed between these ECs. Specifically, ECs limit BBB permeability by inhibiting paracellular channels and non-specific transcellular transport. TJs consist of occludin, claudins, tricellulins, and other proteins^250^; a decrease in TJ protein level during ischemia increases BBB permeability. During cerebral I/R, the upregulation of MMP9 expression contributes to the degradation of TJ proteins between brain ECs, further damaging the BBB. Overall, this process is negatively regulated by Wnt/β-catenin signaling.^10,251^

NHE1 protein promotes the H^+^-Na^+^ exchange in astrocytes. During cerebral ischemia, NHE1 is activated, resulting in Na^+^ overload and cell swelling in astrocytes; this eliminates the corresponding BBB maintenance function of astrocytes, leading to BBB damage.^252^ Song et al. demonstrated that Wnt7a/7b expression was upregulated and the BBB was repaired, after knocking out the *Nhe1* gene in the astrocytes of cerebral ischemic mice. Astrocytes with the *NHE1* knockout can activate Wnt/β-catenin signaling, thereby exhibiting a protective role in cerebral ischemia. Furthermore, Song et al. demonstrated that the upregulation of Wnt7a/7b expression in *NHE1*^*−/−*^ astrocytes promotes Wnt/β-catenin signaling activation and facilitates BBB repair.^253^

### Wnt pathways during renal I/R injury

Renal I/R injury is an inevitable and serious complication following renal transplantation^40^ and the main factor that promotes acute kidney injury (AKI) and reduces long-term renal graft survival rates.^254,255^ In normal adult kidneys, the activity of Wnt/β-catenin signaling is relatively low.^256^ However, when the kidney undergoes damage, Wnt/β-catenin and Wnt/Ca^2+^ signaling is promoted, which further damages the kidney via cell senescence, renal fibrosis, oxidative stress, apoptosis, and ferroptosis pathways, thereby causing AKI or chronic kidney disease (CKD). Overall, this process involves intercellular communication between the renal tubular epithelium and interstitial fibroblasts.

#### Apoptosis

**Fig. 6:**
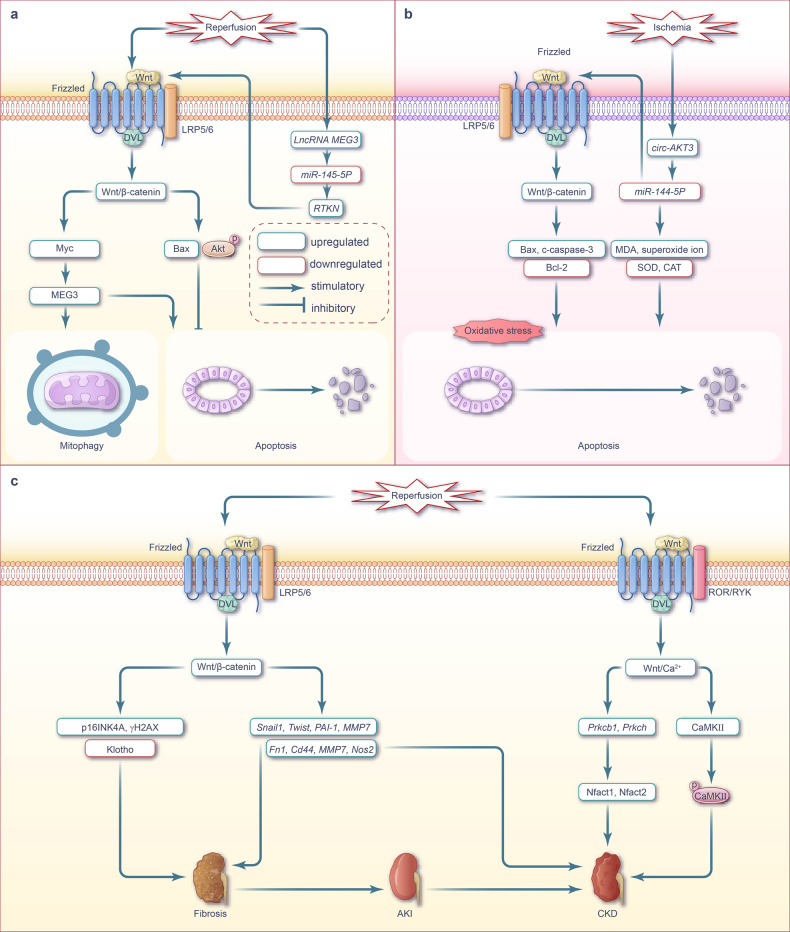
Wnt signaling pathway and targeted therapy during renal I/R injury. **a** Wnt signaling-mediated apoptosis during renal I/R injury. During the renal I/R phase, there is an upregulation of lncRNA *MEG3*, which leads to the activation of Wnt/β-catenin signaling. This activation, in turn, promotes mitophagy and induces apoptosis in renal cells. **b** Wnt signaling-mediated oxidative stress during renal I/R injury. In the renal ischemic phase, there is a downregulation of *miR-144-5p*, which in turn activates the Wnt/β-catenin signaling pathway. This activation leads to increased oxidative stress and apoptosis in renal cells. Additionally, *circ-AKT3* further contributes to the reduction of *miR-144-5p* expression, thereby exacerbating renal cell apoptosis. **c** Wnt signaling-mediated cell senescence and renal fibrosis apoptosis during renal I/R injury. During the phase of renal I/R, the Wnt/β-catenin signaling pathway is activated, leading to the promotion of renal cell apoptosis and the development of fibrosis. Additionally, the activation of Wnt/Ca^2+^ signaling during this process contributes to chronic kidney injury. Note: The pink background represents the ischemic phase, while the cream background represents the reperfusion phase. LRP low-density lipoprotein receptor-related protein, ROR recombinant receptor tyrosine kinase like orphan receptor, RYK receptor tyrosine kinase, MDA malondialdehyde, SOD superoxide dismutase, CAT catalase, CaMKII calmodulin-dependent protein kinase II, AKI acute kidney injury, CKD chronic kidney disease

In renal I/R, the activation of Wnt/β-catenin signaling pathway has been suggested to promote apoptosis, while others have suggested otherwise (Fig. 6a). Liu et al. used HK-2 cells to construct a mouse renal I/R injury in vitro model and demonstrated that the expression of lnc *MEG3* in HK-2 cells was significantly upregulated following I/R injury. The combination of lnc *MEG3* and *miR-145-5p* reduced *miR-145-5p* content, upregulated the expression of its downstream target *RTKN*, activated the Wnt/β-catenin pathway and its downstream effector Myc, promoted the expression of lnc MEG3, and aggravated renal injury.^257^ Alternatively, silencing lnc MEG3 in HK-2 cells with I/R stress inhibited Wnt/β-catenin signaling, Myc expression, mitophagy, and apoptosis, and alleviated renal tubular injury.^257^ However, other evidence indicated that after 1 day of renal I/R-induced AKI, the expression of β-catenin in renal tubular cells was significantly upregulated, which reduced renal tubular cell apoptosis, thereby exerting a protective effect. In vitro experiments have confirmed that Wnt1 activates β-catenin, promotes the phosphorylation of Akt and the expression of survivin, and inhibits the expression of Bax and p53, and therefore activates the anti-apoptotic mechanism and reduces AKI following renal I/R injury.^258^

Taken together, the effects of activating Wnt/β-catenin signaling during renal I/R injury remain controversial, therefore further investigations are warranted. Nonetheless, we are more inclined to accept the findings of the study conducted by Zhou et al. Specifically, Liu et al. only evaluated the role of lncRNA MEG3 using cell experiments; the authors indicated that the Wnt signaling pathway might be only one downstream pathway and did not indicate that Wnt/β-catenin eventually leads to damage. In contrast, Zhou et al. used both in vitro and in vivo experiments to demonstrate the mechanism by which the Wnt/β-catenin pathway protects against early renal I/R injury.

#### Ferroptosis

The Wnt/β-catenin signaling pathway is associated with ferroptosis following renal I/R injury; however, the specific connection between these two mechanisms remains unclear. Ferroptosis aggravates AKI and delays graft function (DGF) following renal I/R injury.^259^ Utilizing LASSO analysis, Wei et al. determined that the emergency response gene activation transformation factor 3 (*ATF3*) was a high-risk gene for ferroptosis-associated DGF during renal I/R injury. ATF3 was reported to be highly expressed within the Wnt/β-catenin pathway and implicated in the regulation of chemokine-associated pathways.^260^ Overall, these results indicate that DGF-associated ferroptosis is linked to Wnt/β-catenin signaling; however, the precise mechanisms remain yet to be elucidated.

#### Oxidative stress

The activation of Wnt/β-catenin signaling during renal I/R promotes oxidative stress (Fig. 6b). This increase in oxidative stress and decrease in antioxidants may be the primary cause of subsequent renal injury. Studies have shown that inhibition of Wnt/β-catenin signaling can reduce the oxidative stress and inflammatory responses mediated by renal I/R injury.^25^

As a non-coding RNA, *miR-144-5p* is involved in the regulation of gene expression after transcription. Xu et al. demonstrated that in hypoxic rat HK2 cells, *miR-144-5p* expression was downregulated, leading to activation of Wnt/β-catenin signaling. This activation resulted in increased expression of Bax and caspase 3, along with reduced Bcl-2 expression, ultimately leading to cell injury and apoptosis. *Circ-AKT3* has been shown to be an effective *miR-144-5p* sponge that can further reduce the expression of *miR-144-5p* following renal I/R injury in rats. Consequently, this *miR-144-5p* inhibition significantly increases the malondialdehyde and superoxide ion content, as well as reduced activity of superoxide dismutase (SOD) and catalase (CAT). Overall, this induces in oxidative stress, apoptosis, and the aggravation of renal injury.^261^ Therefore, c*irc-AKT3* causes apoptosis and aggravates renal injury by activating Wnt/β-catenin signaling and increasing oxidative stress.^261^

#### Cell senescence and renal fibrosis

The Wnt/β-catenin signaling pathway is activated after renal I/R injury and promotes senescence and renal fibrosis. Wnt/Ca^2+^ signaling pathway activation also results in the promotion of cell senescence and renal fibrosis (Fig. 6c). Cell growth arrest, DNA double-strand structural damage, and the accumulation of senescence-associated proteins are the main characteristics of cell senescence. The accumulation of senescence-associated proteins is predominantly mediated by p16INK4A-Rb and ARF-p53-p21 signaling.^262^

The Wnt signaling pathway is an important participant in renal fibrosis^53^ that accelerates cell senescence, especially by regulating the DNA double-stranded structure and the balance between cellular senescence proteins and anti-aging proteins, which ultimately leads to renal fibrosis. With the aggravation of renal I/R injury, sustained activation of Wnt/β-catenin signaling promotes the transcription of downstream fibrogenic genes, including *SNAI1*, *TWIST*, *PAI1*, and *MMP7*, which can induce renal fibrosis and accelerate the progression of AKI to CKD.^263^

Luo et al. evaluated a CKD mouse model with unilateral I/R and established that Wnt9a levels increased slightly 1 day after severe I/R injury and significantly increased 3 days later. These Wnt9a levels were positively correlated with an increase in p16INK4A and γΗ2ΑX (a selective marker of DNA double-strand break) and a decrease in the anti-aging protein Klotho (a marker of renal tubular injury and CKD).^264^ Increased Wnt9a ligand expression activates Wnt/β-catenin signaling, which promotes the expression of downstream profibrogenic and transcriptional targets that exacerbate renal fibrosis.^264–268^

In a kidney transplantation rat model, Toerne et al. observed glomerular disease, impaired tubulointerstitium and renal fibrosis in transplanted rat kidneys, similar to chronic allograft injury sites after human kidney transplantation.^269^ Sun et al. conducted a prospective multicenter controlled study and reported that allogeneic mesenchymal stem cells significantly reduced DGF, acute rejection, and prolong long-term survival in renal transplantation.^270^ Furthermore, with the progression of renal injury, the expression levels of canonical Wnt pathway–related genes *Fn1, Cd44*, *Mmp7*, and *Nos2* were upregulated, and so were those of the Wnt/Ca^2+^ pathway–related genes *Prkcb1*, *Prkch*, *Nfact1_pred*, and *Nfact2*. Additionally, the expression of CaMKII protein in monocyte infiltration increased, and the phosphorylation of this protein increased significantly with the development of fibrosis. The authors hypothesized that this morphological change was related to the activation of canonical Wnt signaling and non-canonical Wnt/Ca^2+^ signaling in the transplanted kidney, contributing to chronic kidney injury following renal transplantation.^269^ However, the role of the Wnt/PCP pathway in cell senescence and renal fibrosis following renal transplantation is yet to be determined.

### Wnt pathways during hepatic I/R injury

Hepatic I/R injury is a pathophysiological event that occurs following liver surgery or transplantation and profoundly affects the prognosis of liver function. The degree of liver I/R injury is influenced by temperature, ischemic time, and range. The injury of liver cells caused by warm ischemia is more severe than that caused by cold ischemia, whereas the injury to liver sinusoidal ECs is the opposite.^271^ Under physiological conditions, the Wnt signaling pathway regulates hepatocyte functions such as proliferation, survival, metabolism, regeneration, liver homeostasis, and cell–cell adhesion.^272^ In general, the pathological process of hepatic I/R injury involves damage to sinusoidal ECs, liver cells, hepatic stellate, and other cells. The Wnt signaling pathway participates in the regulation of apoptosis, necrosis, inflammatory responses, oxidative stress, and proliferation during hepatic I/R injury.

#### Inflammation and apoptosis

**Fig. 7:**
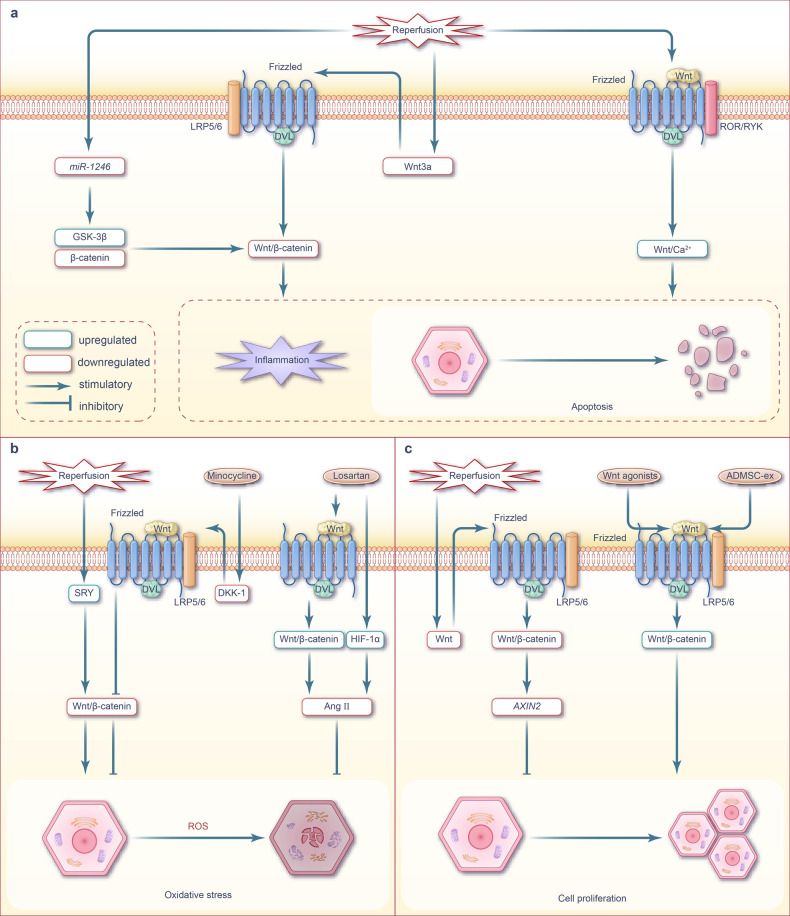
Wnt signaling pathway and targeted therapy during hepatic I/R injury. **a** Wnt signaling-mediated inflammation and apoptosis during hepatic I/R injury. During hepatic I/R injury, the Wnt/β-catenin signaling pathway is suppressed, leading to the promotion of liver inflammation and apoptosis. Concurrently, Wnt/Ca^2+^ signaling is activated, exacerbating cell apoptosis. **b** Wnt signaling-mediated oxidative stress during hepatic I/R injury. During hepatic I/R injury, the Wnt/β-catenin signaling is inhibited, resulting in the aggravation of oxidative stress. However, treatment with minocycline and losartan treatment can alleviate intracellular oxidative stress by activating the Wnt/β-catenin signaling pathway. **c** Wnt signaling-mediated cell proliferation during hepatic I/R injury. During hepatic I/R injury, the Wnt/β-catenin signaling is inhibited, leading to a decrease in the transcription of the downstream target gene *AXIN2* and subsequent inhibition of cell proliferation. However, the activation of Wnt/β-catenin signaling through the use of Wnt agonists and ADMSCs-ex can promote cell proliferation in this context. Note: the cream background represents the reperfusion phase. LRP low-density lipoprotein receptor-related protein, ROR recombinant receptor tyrosine kinase like orphan receptor, RYK receptor tyrosine kinase, DKK-1 Dickkopf-1, HIF-1α hypoxia-inducible factor-1α, ADMSC-ex adipose-derived mesenchymal stem cell exosomes

During hepatic I/R injury, Wnt/β-catenin signaling is inhibited, aggravating inflammation and apoptosis, while the Wnt/Ca^2+^ signaling pathway is activated and further promotes apoptosis (Fig. 7a). During hepatic I/R injury, sinusoidal ECs and hepatocytes release DAMPS, which trigger an inflammatory response.^273^ In hypoxia and H/R liver cells, both Wnt/β-catenin signaling and HIF1α signaling were inhibited,^274^ which synergistically aggravated liver oxidative stress and promoted apoptosis.^274^ Liu et al. determined that in the liver tissue of non-lethal hepatic I/R mice and H/R hepatocytes, downregulation of Wnt3a and β-catenin expression, and inhibition of Wnt/β-catenin signaling aggravated liver apoptosis, necrosis, and the inflammatory response.^275^ Xie et al. established that miR-1246 expression was significantly downregulated in I/R mouse liver tissues and H/R-treated LO2 cells.^276^ By targeting the negative regulation of GSK-3β, GSK-3β protein levels could be significantly upregulated, while β-catenin expression downregulated, which inhibited the Wnt/β-catenin pathway and upregulated hepatocyte apoptosis.^276^

The non-canonical Wnt/Ca^2+^ signaling pathway is also involved in the regulation of apoptosis during hepatic I/R injury. Sakon et al. found elevated calpain activity in allograft biopsy specimens collected from patients with poor function after liver transplantation.^277^ This increase in calpain activity corresponded to a similar increase in Ca^2+^ concentration during liver I/R.^277^ Hu et al. demonstrated that activation of Wnt/Ca^2+^ signaling promoted apoptosis in normal rat hepatocytes BRL-3A under conditions of hypoxia-reoxygenation.^278^ Additionally, in an I/R injury model of rat H9c2 cells, Zhou et al. found that upregulation of the Wnt5/Frizzled-2 pathway in liver tissue led to increased Ca^2+^ activity and apoptosis.^103^

Targeting the upstream elements of Wnt and its corresponding signaling pathway could alleviate hepatocyte inflammation and inhibit apoptosis following hepatic I/R (Fig. 7a). Terlipressin, a synthetic antidiuretic hormone analog, can improve the survival of patients with advanced cirrhosis.^279,280^ Terlipressin selectively binds to the V1 receptor in the human liver.^279,280^ Therefore, terlipressin treatment activates the Wnt/β-catenin/FoxO3a/AKT pathway by upregulating V1 receptor, which significantly improves I/R-induced hepatocyte apoptosis, necrosis, and inflammation, and ultimately protects the liver.^275^ Kohler et al. conducted a randomized, double-blind, placebo-controlled trial involving 150 patients who underwent selective large hepatectomy and determined that perioperative terlipressin did not affect the endpoint of liver-specific complications, but did significantly prevent postoperative liver function degradation.^281^ Similar findings were also observed by Hong.^282^ Alternatively, intravenous injection of human umbilical cord blood mesenchymal stem cell-derived exosomes into the portal vein of hepatic I/R mice upregulated the expression of miR-1246 and activate the Wnt/β-catenin pathway by targeting and negatively regulating GSK-3β; therefore, this treatment exerts anti-apoptotic effects and alleviates hepatic I/R injury.^276^ Agmatine (AGM) is an endogenous polyamine that confers a protective effect on I/R injury in the brain, kidney, heart, and other tissues and organs.^283–286^ Intraperitoneal injection of AGM has been observed to inhibit inflammation and apoptosis following hepatic I/R in mice via activation of the Wnt/β-catenin signaling pathway.^287^ Alternatively, transfection of Frizzled-2 siRNA into rat liver BRL-3A cells silenced *Frizzled-2* gene expression; this reduced the intracellular Ca^2+^ concentration increase induced by H/R, inhibited Wnt/Ca^2+^ signaling, and ultimately reduced cytotoxicity and apoptosis.^278^

#### Oxidative stress

The inhibition of the Wnt/β-catenin signaling pathway during hepatic I/R contributes to oxidative stress in hepatocytes (Fig. 7b). Dong et al. established that the postoperative transaminase levels, total incidence, and incidence of liver failure in 1,267 male patients undergoing hepatectomy were significantly higher than those in 508 female patients^288^ and concluded that males are more prone to I/R injury than females.^288^ *SRY*, a mammalian sex determination gene,^289^ is significantly upregulated in hepatic I/R injury.^288^ Following hepatic I/R, upregulated SRY interacts with GSK-3β and β-catenin, promotes the phosphorylation and degradation of β-catenin, and inhibits Wnt/β-catenin signaling, which aggravates liver inflammation, oxidative stress, and cell necrosis.^288^ Furthermore, dysregulation of Wnt3a, β-catenin, and HIF-1α, as well as antioxidant enzyme activities (Mn-SOD, Cu/Zn-SOD, glutathione and CAT), synergistically promotes oxidative stress by inhibiting the Wnt/β-catenin and HIF-1α signaling pathways in the liver during I/R injury.^290^

Overall, these findings suggest that activation of the Wnt/β-catenin signaling pathway reduces oxidative stress following hepatic I/R (Fig. 7b). Administration of Mino, an antibiotic with anti-inflammatory, anti-apoptotic, and anti-oxidative properties,^291^ reduced DKK1 protein expression, increased β-catenin protein expression, and activated the Wnt/β-catenin signaling pathway, thereby protecting the liver from I/R injury.^292^ Ang II, the main effector peptide of the renin-angiotensin system, can stimulate hepatic stellate cells and Kupffer cells to exacerbate oxidative stress.^293–296^ The activity of Ang II in the liver is primarily mediated by AT 1Rs (Ang II type I receptor).^293–296^ Similarly, losartan, an AT 1Rs antagonist, protects against I/R injury by upregulating Wnt/β-catenin and HIF-1α signaling,^297^ mitigating oxidative stress and safeguarding the liver.^290^

#### Cell proliferation

Hepatic I/R inhibits hepatocyte proliferation and liver injury repair by dampening the Wnt/β-catenin signaling pathway (Fig. 7c). This signaling cascade plays a pivotal role in hepatocyte proliferation and liver regeneration.^272^ The downregulation of Wnt/β-catenin signaling during hepatic I/R curtails the transcription of downstream target gene *Axin2*, leading to a subsequent decline in hepatocyte proliferation and liver injury repair.^298^ Fortunately, the use of Wnt agonists can counteract this inhibition and promote the upregulation of the Wnt/β-catenin cascade, thereby stimulating hepatocyte proliferation and facilitating the repair of liver injury.^298^ Sun et al. demonstrated that liver regeneration mechanisms were exhibited in hepatocytes throughout the entire organ after partial hepatectomy, and the upregulation of Axin2 specifically contributed to this hepatocyte proliferation.^299^ Axin2 interacts with β-catenin/CTNNB1, an intracellular scaffold protein,^300^ although the precise dependence of Axin2 upregulation on Wnt/β-catenin signaling post-partial hepatectomy remains nebulous.^299^ Nonetheless, following I/R combined with hepatectomy in rats, the downregulation of Wnt2, β-catenin, and cyclin D1, as well as the inhibition of the Wnt/β-catenin pathway, hindered liver cell proliferation and regeneration.^301^

Collectively, these findings underscore the significance of upregulating the Wnt/β-catenin signaling pathway to drive liver cell proliferation following hepatic I/R (Fig. 7c). Intraperitoneal injection of Wnt agonists in rats undergoing hepatic I/R can effectively elevate Wnt/β-catenin signaling, resulting in a remarkable increase in hepatocyte proliferation while concurrently reducing apoptosis and necrosis.^298^ Additionally, intravenous injection of ADMSC-ex^301^ into hepatic I/R rats can activate the Wnt/β-catenin signaling pathway, upregulate the expression of the regeneration-related factors such as Cyclin D1 and VEGF, and foster the proliferation and regeneration of liver cells.^301^

## Crosstalk between Wnt signaling and other I/R-associated pathways

### Notch signaling

The Notch signaling pathway, a highly evolutionarily conserved pathway involved in embryonic development and tissue injury repair,^302,303^ is an important pathway in the development and prognosis of organ I/R.^62,304–308^ The crosstalk between the Notch and Wnt signaling pathways contributes to various cellular processes, including cell proliferation, apoptosis, fibrosis, tumorigenesis, and metastasis.^309–312^ Studies on cardiac and cerebral I/R have revealed that the interaction between the Notch and Wnt signaling pathways coordinates the regulation of pathological processes such as apoptosis and inflammatory responses. However, whether such crosstalk occurs during liver or kidney I/R remains to be investigated.

The Notch signaling pathway is composed of both canonical and non-canonical pathways. The canonical Notch pathway consists of five Notch ligands, namely jagged1, jagged2, and Delta-like1–4, and four receptors, namely Notch1–4. The canonical Notch signaling pathway is activated when Notch ligands bind to Notch receptors, which then release corresponding intracellular domains; these intracellular domains then translocate to the nucleus and bind to the transcription factor cardiolipin synthetic lecithin (CSL), which induces the transcription of downstream target genes.^313^ Meanwhile, the non-canonical Notch pathway is independent of CSL and instead regulates the transcription of target genes by synergizing with multiple signaling pathways.^302^

**Fig. 8:**
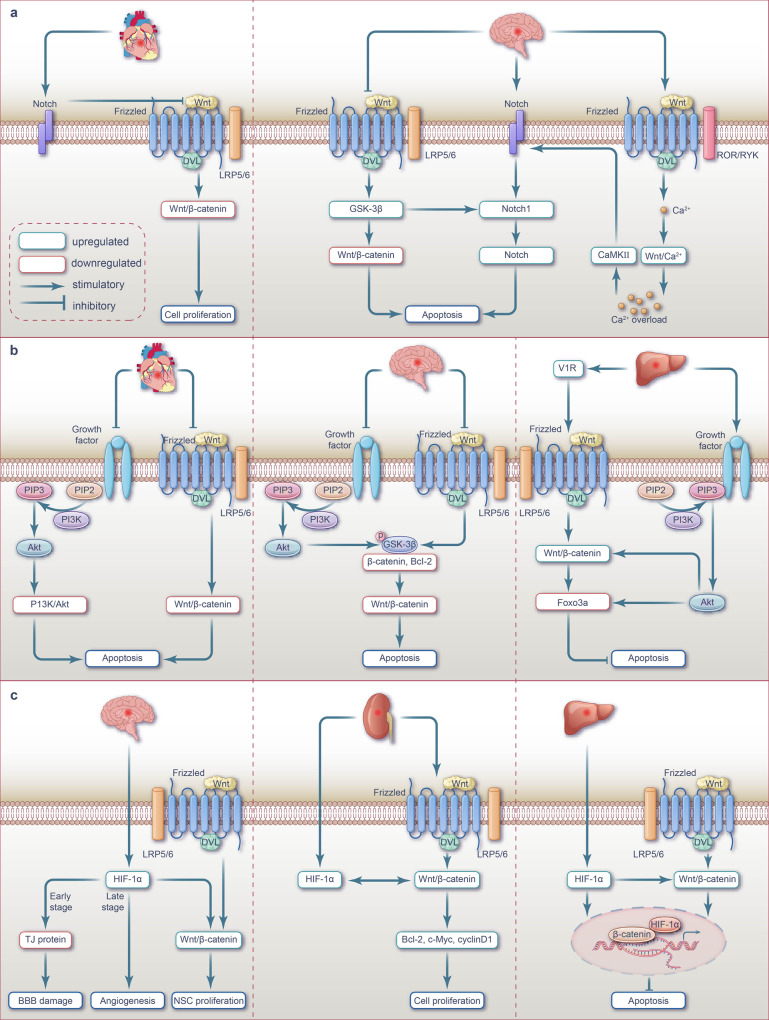
Interplay of Wnt, Notch, and PI3K/Akt signaling pathways during I/R Injury: Insights from Crosstalk Mechanisms. **a** Crosstalk between Wnt and Notch signaling pathway during I/R injury. During zebrafish myocardial I/R injury, the activated Notch signaling inhibites the Wnt signaling transduction and restored the proliferation ability of certain cardiomyocytes. In the process of cerebral ischemia phase, Wnt/β-catenin signaling is inhibited while Notch signaling is activated. These two signals crosstalk through GSK-3β to promote apoptosis. Additionally, the Wnt/Ca^2+^ signaling pathway is concurrently upregulated, which crosstalks with Notch signaling to enhance the Notch signaling activity. **b** Crosstalk between Wnt and PI3K/Akt signaling pathway during I/R injury. During myocardial I/R injury, both PI3K/Akt and Wnt/β-catenin signaling pathways are downregulated. This downregulation inhibits the crosstalk between these pathways, ultimately resulting in cardiomyocyte apoptosis and left ventricular dysfunction. Similarly, during cerebral I/R injury, both PI3K/Akt and Wnt/β-catenin signal pathways are downregulated. In this context, these pathways crosstalk through GSK-3β to promote cell apoptosis, thereby contributing to the pathophysiology of cerebral I/R injury. During hepatic I/R injury, the expression of antidiuretic hormone receptor 1 (V1R) in hepatocytes is upregulated. This upregulation plays a protective role by activating Wnt/β-catenin/FoxO3a/Akt pathway, and then conceals apoptosis induced by FoxO3a activation. **c** Crosstalk between Wnt and HIF-1α signaling pathway during ischemia or I/R injury. In the context of cerebral ischemia and hypoxia, the increased activity of the HIF-1α signaling pathway plays a role in enhancing the proliferation and neuronal differentiation of neural stem cells by activating the Wnt/β-catenin signaling pathway. During the early stages of the pathological process, HIF-1α disrupts TJ proteins, which subsequently leads to an elevated permeability of the BBB. As the pathological process progresses to the later stages, HIF-1α promotes angiogenesis. In the model AKI induced by H/R, the activation of Wnt/β-catenin signaling pathway has been shown to enhance the protective effect of HIF, Simultaneously, HIF increases the expression of β-catenin and its downstream target genes. This interaction and crosstalk between HIF and Wnt/β-catenin signaling pathway contribute to early renal repair after AKI. In context of hepatic hypoxia or H/R, HIF-1α has the ability to competitively bind to β-catenin, leading to enhanced HIF-1α signaling transduction. This interaction serves to reduce apoptosis and promote cell survival in hepatic cells. LRP low-density lipoprotein receptor-related protein, ROR recombinant receptor tyrosine kinase like orphan receptor, RYK receptor tyrosine kinase, CaMKII calmodulin-dependent protein kinase II, PI3K/Akt phosphoinositide-3 kinase/protein kinase B, BBB blood-brain barrier, TJ protein tight junction protein, AKI acute kidney injury, I/R ischemia–reperfusion, V1R antidiuretic hormone receptor 1, NSC neural stem cells

During myocardial ischemia and I/R injury, the Notch signaling pathway undergoes activation. This activation contributes to the reduction of cardiomyocyte death, the decrease in infarct volume, and the improvement of cardiac function.^314,315^ Zebrafish have the remarkable ability to completely regenerate damaged myocardium by enabling the proliferation of preserved cardiomyocytes. In a study using zebrafish model of endocardial injury, Zhao et al. discovered that the activated Notch signaling could inhibit Wnt signaling in the injured heart. Additionally, the authors observed that injecting the Wnt inhibitor IWR-1-endo into a Notch-deficient zebrafish heart model could partially restore the corresponding proliferation of cardiomyocytes (Fig. 8a). As a result, the authors proposed an antagonistic relationship between Notch signaling and Wnt signaling during cardiac repair.^316,317^ Similarly, during cerebral ischemia and I/R, the Notch signaling is activated. The activation of Notch1 signaling promotes the proliferation of neural stem cells and inhibits apoptosis,^318,319^ while activation of the Notch3 signaling pathway facilitates vascular EC differentiation. This leads to improved vascular adaptation to hypoxic-ischemic conditions and subsequently reduces neural injury.^320,321^ However, Notch signaling has also been reported to exhibit neurotoxic effects during ischemic stroke, which includes inducing apoptosis, activating microglia, and promoting inflammatory cell infiltration.^21,322^ In the neonatal rat cerebral ischemia model, the Notch signaling pathway was activated while the Wnt/β-catenin pathway was inhibited, and these two signals synergistically promoted apoptosis through crosstalk involving GSK-3β^323^ (Fig. 8a).

These findings suggest intermediate molecules in both the Wnt and Notch signaling pathways as potential targets for the effective treatment of organ I/R injury. Treatment with the GSK-3β inhibitor TWS119 showed simultaneous effects of upregulating the Wnt/β-catenin signaling pathway and inhibiting the Notch signaling pathway. This dual modulation results in synaptic proteins and reduced apoptosis in neonatal rats with cerebral ischemia.^323^ In a rat model of brain I/R injury, treatment with the traditional Chinese medicine (TCM) L-Borneolum during the recovery period inhibited the Wnt3a/β-catenin and Notch-1 signaling pathways. This treatment approach effectively increased cerebral blood flow, promoted the differentiation of astrocytes into neurons in the striatum region, and alleviated cerebral infarction and brain atrophy.^324^ Additionally, in a brain microvascular endothelial cell injury model induced by OGD/R, treatment with Zhongfenggao activated the Notch and Wnt signaling pathways. This activation resulted in a significant increase in VEGF expression and promoted angiogenesis. Based on these findings, the authors proposed that Zhongfenggao holds potential as a therapeutic candidate for the treatment of brain I/R injury.^325^

### PI3K/Akt signaling

The PI3K/Akt signaling pathway, named after its key components, phosphatidylinositol 3-kinase (PI3K) and AKT (also known as protein kinase B, PKB), plays a crucial role in regulating cell proliferation, survival, and apoptotic processes.^326^ Crosstalk between the PI3K/Akt and Wnt signaling pathways influences osteoblast proliferation, differentiation, and cancer development.^327^ The PI3K/Akt/Wnt signaling crosstalk has been identified as a critical pathological process in I/R injury of the heart, brain, and liver, impacting apoptosis and angiogenesis. However, our current understanding regarding PI3K/Akt/Wnt crosstalk in renal I/R remains limited.

PI3K has three isoforms, namely types I–III. PI3K activation induces the conversion of phosphatidylinositol 3,4-bisphosphate to phosphatidylinositol 3,4,5-trisphosphate. Phosphatidylinositol 3,4,5-trisphosphate is a secondary messenger that promotes the translocation of the downstream protein AKT to the cell membrane. AKT is a serine/threonine protein kinase, with several isoforms: AKT1, AKT2, and AKT3. AKT phosphorylation activation results in the translocation of AKT from the cell membrane to the cytoplasm or nucleus to allow further regulation of downstream targets such as mTOR, NF-κB, and Bad.^328–331^

In a porcine model of chronic myocardial I/R, the inhibition of calpain activity resulted in the upregulation of both PI3K and Wnt/β-catenin pathways. As a result, there was an increase in blood vessel density observed in both the ischemic and non-ischemic myocardial tissue. Ultimately, this enhanced vascularization contributed to the survival of cardiomyocytes.^332^ In H/R-treated rat cardiomyocytes, overexpression of Akt1 and Wnt11 reduced cardiomyocyte apoptosis. However, this protective effect could be blocked by neutralizing antibodies against Wnt11 or inhibitors of Akt1^333^ (Fig. 8b).

This suggests that the synergistic mechanism of these two signaling pathways also plays a role in I/R injury of the brain and liver (Fig. 8b). The PI3K/Akt pathway acts as an inherent protective mechanism by enhancing Bcl-2 expression, which in turn exerts an anti-apoptotic effect.^334^ The interaction between PI3K/Akt and Wnt/β-catenin signaling occurs through GSK-3β. Activation of PI3K/Akt signaling pathway leads to phosphorylation and inactivation of GSK-3β,^335^ subsequently activating Wnt/β-catenin signaling^336^ and exerting a protective effect against cerebral I/R injury^337^ (Fig. 8b). FoxO3a is in the downstream of the PI3K/AKT pathway. Liu et al. conducted a study using a mouse model of hepatic I/R and found that activating the Wnt/β-catenin and Akt pathway had a mitigating effect on liver injury resulting from oxidative stress following hepatic I/R. Additionally, they observed that this activation effectively masked apoptotic events induced by elevated FoxO3a level.^275^ These findings highlight the potential of the Wnt/β-catenin and Akt pathway in protecting against liver damage and suppressing apoptotic processes in the context of hepatic I/R.^275^

As the PI3K/AKT/GSK-3β signaling pathway is positioned upstream of Wnt/β-catenin signaling; therapeutics that activate PI3K/AKT/GSK-3β, such as dexmedetomidine or eugenol, have the potential to simultaneously activate Wnt/β-catenin signaling. Therefore, these therapeutic agents are promising strategies for treating organ I/R injury.^338,339^ For instance, in a cerebral ischemia rat model, the administration of dexmedetomidine increased neuronal survival and reduced cerebral infarct size.^338^ Similarly, treatment with XQ-1H demonstrated the ability to promote angiogenesis and to restore neurological function following ischemic stroke.^340^ Lastly, when administered orally for 30 days, Phyllanthus emblica mitigated myocardial damage resulting from I/R in rats undergoing cardiac I/R surgery.^339^

### HIF-1α signaling

The hypoxia-inducible factor-1α (HIF-1α) signaling pathway is responsible for conferring adaptation to hypoxic conditions and plays a crucial role in angiogenesis, oxidative stress, and cell metabolism under hypoxic conditions.^341,342^ Notably, there is a significant crosstalk between the HIF-1α and Wnt signaling pathways, which jointly regulate processes such as osteogenesis, angiogenesis, and the development and migration of various cancers.^343–345^ Recent studies have elucidated the involvement of the HIF-1α and Wnt signaling pathway crosstalk in the regulation in regulation of cell proliferation differentiation, BBB permeability, and apoptosis during I/R injury affecting the brain, liver, kidney, and other organs.

HIF-1 is a member of the HIF family and consists of the HIF-1α and HIF-1β subunits. HIF-1α is an oxygen-dependent protein that exhibits a short half-life under high oxygen conditions and is rapidly degraded by proteasomes. It is, however, stable under hypoxic conditions; under such conditions, HIF-1 translocates to the nucleus, co-polymerizes with HIF-1β to form a heterodimer, and binds to hypoxic response elements to promote the expression of the downstream transcription factors VEGF and glucose transporter-1.^346^

Research has shown that under conditions of cerebral ischemia and hypoxia, the HIF-1α signaling pathway is activated.^347^ The upregulated HIF-1α signaling pathway can induce the activation of the Wnt/β-catenin signaling pathway, promoting the proliferation of neural stem cells and neuronal differentiation^226^ (Fig. 8c). It can also disrupt TJ proteins, leading to increased permeability of the BBB and upregulation of the target gene *VEGF*, thereby exacerbating vascular leakage.^347^ Based on the dual role of *VEGF*, the elevation of *VEGF* promotes angiogenesis during the late stage of ischemia and hypoxia^240^ (Fig. 8c). In contrast, a study on cerebral ischemia revealed that the HIF-1α/VEGF signaling pathway was downregulated during the treatment period; this suppression, in turn, activated the Wnt/β-catenin signaling pathway, thereby improving brain microenvironment in rats with MCAO.^348^ The conflicting outcomes of these findings can be attributed to the intricate interplay and complexity of mechanisms between these signaling pathways.

Similarly, the crosstalk between the HIF-1α and Wnt/β-catenin signaling pathways in liver I/R injury synergistically reduces oxidative stress following hepatic I/R^290^(Fig. 8c). After hepatic ischemia or reperfusion, the expression level of β-catenin regulates the activity of the HIF-1α signal,^274^ and the lack of β-catenin leads to the inhibition of HIF-1α signal transduction after hepatocyte hypoxia.^274^ Under hypoxia or H/R conditions, HIF-1α can competitively inhibit the interaction between TCF4 and β-catenin, which enhances HIF-1α signal transduction, reduces apoptosis, and promotes cell survival.^274,349^ Furthermore, Crosstalk between HIF and Wnt/β-catenin signaling pathway has also been reported in renal I/R. Xu et al. demonstrated that in a cellular model of H/R injury, activated Wnt/β-catenin and HIF signaling pathways promoted each other and enhanced the expression of downstream target genes of Wnt/β-catenin signaling pathway, synergistically promoted early renal repair following AKI^350^ (Fig. 8c).

Collective evidence suggests that that the crosstalk between the HIF-1α and Wnt/β-catenin signaling pathways plays a crucial role in mitigating organ damage caused by organ I/R injury. This beneficial effect has been observed in various organs, including the brain, kidneys, and liver. In a hypoxic environment, peroxynitrite production activates HIF-1, which is associated with the Wnt/β-catenin signaling pathway, leading to the promotion of neural stem cell proliferation, self-renewal, and neuronal differentiation.^226^ Moreover, HIF activation promotes cell proliferation and inhibits apoptosis in renal IR-induced AKI, and these effects can be reversed by treatment with the β-catenin inhibitor IWR-1-endo, indicating interaction between HIF and the Wnt/β-catenin signaling pathway in providing renal protection.^350^ Additionally, the angiotensin II type 1 receptor antagonist losartan enhances HIF-1α and Wnt/β-catenin signaling, thereby alleviating liver I/R damage^290^ by restoring HIF-1α and β-catenin content. These findings highlight the therapeutic potential of modulating the crosstalk between HIF-1α and Wnt/β-catenin signaling pathways as a strategy to protect organs from I/R injury and promote their recovery.

### TGF-β signaling

The TGF-β signaling pathway, comprising both Smad-dependent and Smad-independent pathways, plays a crucial regulatory role in early embryonic development as well as in disease states such as fibrosis and cancer.^351^ Notably, crosstalk has been observed between the TGF-β and Wnt signaling pathways, where Smad3 forms a complex with β-catenin, inhibiting its degradation and facilitating its nuclear translocation, thus activating the Wnt/β-catenin signaling pathway.^352^ This crosstalk has been implicated in heart, brain, liver, and kidney I/R injury, contributing to pathological processes such as apoptosis and fibrosis.

TβR-I–III are the three types of TGF-β receptors. Smad family proteins are downstream of the TGF-β signaling pathway, and include receptor-regulated Smads, common pathway Smads, and inhibitory Smads. In the Smad-dependent pathway, the binding of TGF-β to its receptors leads to phosphorylation of receptor-regulated Smads. Phosphorylated R-mads then forms complexes with Co-Smads and translocates to the nucleus to further regulate the transcription of downstream target genes. Alternatively, the Smad-independent pathway utilizes TGF-β to activate a variety of downstream cascades, such as mitogen-activated protein kinase (MAPK), PI3K/AKT, Rho-like, and JNK signaling pathways,^351,353^ but not Smad signaling.

**Fig. 9:**
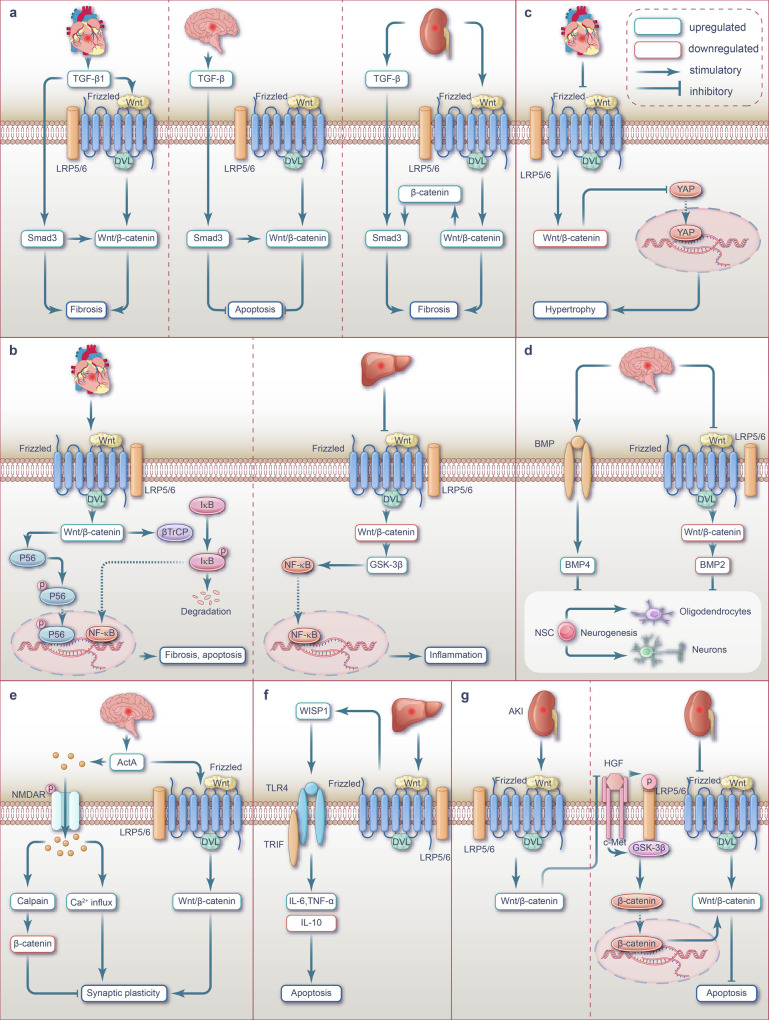
Interplay between Wnt, TGF-β, NF-κB, Hippo-YAP, BMP, NMDAR-Ca^**2+**^-ActA, TLR4/TRIF and HGF/c-Met signaling pathways during ischemia or I/R injury. **a** Crosstalk between Wnt and TGF-β signaling pathway during ischemia injury. After myocardial ischemia, there is an increased expression of TGF-β1, which subsequently activates the Wnt/β-catenin signaling pathway. These two pathways work synergistically to promote the process of myocardial fibrosis. During cerebral ischemia and I/R injury, the TGF-β/Smad signaling pathway is activated and collaborates with the Wnt/β-catenin signaling pathway to reduce the apoptosis in cortical neuronal caused by cerebral ischemia. Similarly, in renal I/R injury, the TGF-β signaling pathway is activated and interacts with the Wnt/β-catenin signaling pathway, contributing to the progression of fibrosis. During this process, β-catenin functions as a transcription cofactor of Smad3, promoting the transcription of downstream target genes and facilitating EMT. **b** Crosstalk between Wnt and NF-κB signaling pathway during ischemia or I/R injury. During myocardial ischemia, the upregulated Wnt/β-catenin signaling pathway facilitates the activation of NF-κB signaling pathway by promoting nuclear translocation of p65, this activation induces the migration of cardiac fibroblasts. Additionally, the activated Wnt/β-catenin signaling promotes the degradation of phosphorylated IκB mediated through β-TrCP, subsequently promoting the nuclear translocation of NF-κB, leading to myocardial fibrosis and apoptosis. In contrast, In the process of liver I/R injury, the Wnt/β-catenin signaling pathway is inhibited, while NF-κB signaling pathway is activated. The transcription of NF-κB is positively regulated by GSK-3β, which promoted inflammation. the Wnt/β-catenin signaling pathway is inhibited, while the NF-κB signaling pathway is activated. The nuclear translocation of NF-κB is positively regulated by GSK-3β, thereby promoting inflammation. **c** Crosstalk between Wnt and Hippo-YAP signaling pathway during I/R injury. Following myocardial I/R injury, the Wnt/β-catenin signaling is downregulated, leading to the inhibition of YAP1 transcription. Consequently, the activity of the Hippo-YAP signaling pathway is suppressed. This cooperative effect between the two signaling pathways contributes to the promotion of myocardial hypertrophy. **d** Crosstalk between Wnt and BMP signaling pathway during ischemia injury. During cerebral hypoxia, there is an upregulation in the expression of BMP4, while the Wnt/β-catenin signaling is inhibited. Inhibition of Wnt/β-catenin downregulates BMP2 protein expression; thus, the suppressed Wnt/β-catenin signaling and BMP signaling synergistically inhibit the differentiation of neural stem cells into neurons and oligodendrocytes, thereby aggravating cerebral ischemic injury. **e** Crosstalk between Wnt and NMDAR-Ca^**2+**^-ActA signaling pathway during I/R injury. During cerebral ischemia, ActA expression is upregulated, which leads to the Ca^2+^ influx during synaptic transmission and can also activate the Wnt/β-catenin signaling pathway, synergistically regulating synaptic plasticity. However, Ca^2+^ influx mediated by NMDAR activation can also trigger the activation of calpain. This activation of calpain subsequently induces cleavage of β-catenin, resulting in a decrease in synaptic stability. **f** Crosstalk between Wnt and TLR4/TRIF signaling pathway during I/R injury. During hepatic I/R injury, upregulated WISP1 expression activates the TLR4/TRIF signaling pathway, promoting liver injury. **g** Crosstalk between Wnt and HGF/c-Met signaling pathway during I/R injury. During renal I/R injury, activated HGF promotes the phosphorylation of LRP5/6 and plays an anti-apoptotic effect by activating the Wnt/β-catenin signaling pathway. AKI induces an elevation in Wnt protein levels within renal tubular epithelial cells, consequently activating the Wnt/β-catenin signaling pathway. The activated Wnt/β-catenin signaling inhibits the secretion of HGF and HGF/c-Met in renal interstitial fibroblasts. Through this coordinated regulation, both the Wnt/β-catenin signaling pathway and the HGF/c-Met signaling pathway play a role in the modulation of apoptosis processes in the context of AKI. LRP low-density lipoprotein receptor-related protein, TGF-β transforming growth factor-β, NF-κB nuclear factor-κB, YAP Yes-associated protein, TLR Toll-like receptor, HGF/c-Met hepatocyte growth factor receptor/mesenchymal-epithelial transition factor, BMP bone morphogenetic protein, NMDAR N-Methyl-D-Aspartate Receptor, ActA Activin A; I/R ischemia–reperfusion

TGF-β can directly activate the Wnt/β-catenin signaling, while the Wnt/β-catenin signaling pathway helps to stabilize the TGF-β/Smad signaling. Therefore, these two pathways work synergistically to facilitate their respective functions.^354^ Following MI, the crosstalk between the TGF-β and Wnt/β-catenin signaling pathways promotes the progression of myocardial fibrosis^355^ (Fig. 9a).

Similarly, in cases of cerebral ischemia and I/R, activation of TGF-β signaling leads to the activation of Wnt signaling. The combined action of the TGF-β/Smad and Wnt/β-catenin signaling pathways helps to decrease cortical neuron apoptosis resulting from cerebral ischemia, thus reducing brain injury^170,356^ (Fig. 9a). In case of renal I/R injury, the Wnt/β-catenin/TGF-β signaling crosstalk promotes renal fibrosis (Fig. 9a). Chen et al. demonstrated that inhibition of Wnt/β-catenin signaling, TGF-β signaling, and the interaction between these signaling pathways reduced renal fibrosis in renal I/R model in rats.^357^ EMT of renal tubular epithelial cells is an important mechanism underlying renal fibrosis. As a major participant in the EMT process, the TGF-β signaling pathway is activated after renal I/R, and the crosstalk with the Wnt/β-catenin signaling pathway exacerbates this fibrotic process.

The fibrotic effects of TGF-β1 rely on the binding of β-catenin and Smad3 in the nucleus. β-catenin can act as a transcriptional co-factor for Smad3, activating the transcription of downstream target genes and promoting EMT.^358^ Additionally, TGF-β1 can activate the Wnt/β-catenin signaling pathway by inhibiting DKK1 expression, a negative regulator of Wnt signaling.^359^ In pathological conditions such as CKD, increased levels of Wnt9a lead to renal tubular cell senescence and the initiation of TGF-β1 production. TGF-β1 promotes the proliferation of mesenchymal fibroblasts and their transformation into myofibroblasts, while also inducing activated fibroblasts to produce Wnt9a,^268^ thus establishing an intercellular communication loop through different signaling pathways that perpetuates renal fibrosis. The interplay between Wnt and TGF-β signaling pathways has been established in liver fibrosis, but as for its relevance to liver I/R in terms of TGF-β-related treatment approaches, Zhang et al. observed an upregulation of Wnt3a, β-catenin, VEGF, and Cyclin D1, as well as a downregulation of GSK-3β and caspase 3 in a rat model of MCAO following isoflurane injection. This was accompanied by a reduction in infarct size and neuronal apoptosis.^170^ Treatment with TGF-β1 inhibitor LY2157299 before inducing MCAO significantly reduced β-catenin expression in a rat model. Conversely, the Wnt inhibitor DKK-1 did not impact the expression levels of TGF-β1 and Smad3.^170^ These findings suggest that the TGF-β1/Smad3 signaling pathway may have a protective effect by promoting β-catenin expression and reducing apoptosis.^170^ Treatment with Melatonin and Poria acid A may protect the kidneys by inhibiting Smad3 phosphorylation, interfering with β-catenin signaling transduction, suppressing downstream fibrotic targets of the β-catenin pathway, disrupting the interaction between Smad3 and β-catenin, and counteracting the pro-fibrotic effects resulting from the crosstalk between TGF-β/Smad and Wnt/β-catenin signaling pathways in the transition from AKI to CKD.^357^ These findings offer promising therapeutic approaches for attenuating kidney fibrosis.^357^

### NF-κB signaling

NF-κB was initially established as an important transcription factor in the induction of various immune and inflammatory responses.

Nonetheless, the interplay between Wnt and NF-κB signaling pathways has attracted attention due to its regulatory role in inflammation-associated events such as cell proliferation, apoptosis, tumor differentiation, and migration.^79–81^ Moreover, the NF-κB pathway is intricately linked to the progression and prognosis of organ I/R injury.^360–363^ Recent studies have shed light on the crosstalk between Wnt and NF-κB signaling in processes like apoptosis, inflammation, oxidative stress, and fibrosis following ischemic heart injury and liver I/R injury.

The NF-κB family comprises five protein monomers, including p65/RelA, RelB, cRel, p50, and p52, which form homodimers or heterodimers that bind DNA. NF-κB signaling is regulated by two pathways: (1) the NEMO-dependent canonical pathway, where NF-κB acts as a critical modulator of NEMO, and (2) the NEMO-independent non-canonical pathway.^364–366^ In the canonical pathway, inflammatory cytokines, pathogen-associated molecular patterns, or antigen/antibody stimulation trigger IKK phosphorylation, which activates a specific serine on the N-terminus of IκB protein, thereby causing ubiquitination and subsequent proteasomal degradation of IκB.^364–366^ Following the release of IκB, the NF-κB subunit undergoes various post-translational modifications that enable it to bind to specific sites on DNA.^364–366^ The non-canonical pathway, on the other hand, relies on NF-κB-inducing-kinase and IKKα for its activation.^364–366^

Serum Wnt2 and Wnt4 are elevated in patients with acute ischemia.^367^ The upregulation of these Wnt ligands activates Wnt/β-catenin signaling, resulting in p65 nuclear translocation, NF-κB signaling activation, fibroblast migration, and ultimately myocardial fibrosis.^367^ In the inflammatory heart tissue of patients with AMI and obese rats, increased expression of β-catenin induced NF-κB activation and nuclear localization, resulting in myocardial fibrosis and apoptosis^368,369^ (Fig. 9b). The intermediate protein β-transducing repeat-containing protein (βTrCP) plays a crucial role in the crosstalk mechanism between Wnt/β-catenin and NF-κB pathway ^370^. In MI, the activation of Wnt/β-catenin signaling promotes NF-κB nuclear translocation through βTrCP-mediated degradation of phosphorylated IκB.^370–373^ However, there may be antagonism between the Wnt/β-catenin and NF-κB signaling pathways during liver I/R^301^ (Fig. 9b).

The Wnt2/β-catenin signaling pathway associated with liver regeneration is inhibited during liver I/R injury, whereas inflammation-associated NF-κB signaling is activated. However, the crosstalk mechanism between these two pathways during liver I/R injury requires further exploration.^301^ NF-κB is positively regulated by GSK-3β at the transcriptional level, while GSK-3β acts as a negative regulator of the Wnt/β-catenin signaling pathway, potentially explaining this antagonistic relationship.^374^

Based on the crosstalk mechanism of Wnt and NF-κB signaling, Wnt signaling pathway inhibitors, such as Huoxin pill, may simultaneously inhibit NF-κB signaling to alleviate MI.^371^ A similar approach utilizing the Wnt inhibitor DKK1 has been explored in breast cancer treatment to inhibit Wnt/Ca^2+^-CaMKII-NF-κB signaling crosstalk.^375^ In mesenteric I/R-induced liver injury, Mangiferin has been shown to regulate oxidative stress, inflammation, and apoptosis through the Wnt/β-catenin/NF-κB signaling pathway, which involves upregulation of β-catenin and downregulation of NF-κB.^376^ Vitamin D deficiency is a risk factor and potential therapeutic target for AKI, caused by pathological mechanisms such as renal I/R injury.^377^ In a rat model of renal I/R injury, combination therapy with pioglitazone and vitamin D exerted an anti-inflammatory effect by inhibiting the NF-κB signaling pathway.^378^ Moreover, vitamin D has been shown to activate the Wnt4/β-catenin signaling pathway during the early stage of renal I/R and mitigate excessive Wnt4/β-catenin signaling in the later stage to induce renal fibrosis.^378^ However, the precise molecular mechanism of this targeted crosstalk is not yet fully understood. Another approach involves the administration of exosomes derived from fatty mesenchymal stem cells, which inhibit NF-κB phosphorylation while activating the Wnt2/β-catenin signaling pathway.^301^ This intervention has shown promise in reversing the inflammation and pyroptosis caused by hepatic I/R injury, and promoting liver regeneration in hepatic I/R injury.^301^

### Hippo-YAP signaling

The Hippo-YAP signaling pathway, involved in heart development and disease,^379–381^ engages in bidirectional crosstalk with the Wnt signaling pathway, jointly regulating myocardial development and injury under physiological and stress conditions.^379–381^ The Hippo-YAP signaling pathway is an important factor of I/R injury in the heart, brain, kidney, liver, and other organs.

This signaling pathway governs essential processes such as cell proliferation, inflammation, and BBB function following I/R injury.^23,382–385^ Recently, crosstalk between the Hippo-YAP and Wnt signaling pathways has been reported in myocardial I/R injury, in which this crosstalk mechanism regulates myocardial hypertrophy, fibrosis, and inflammation. However, crosstalk between these two pathways has rarely been reported in other organ I/R injuries.

The mammalian Hippo-YAP signaling pathway constitutes a kinase cascade involving core components such as mammalian Ste20-like kinases 1/2, Salvador, large tumor suppressor homolog 1/2, and scaffolding protein MOB domain kinase activator 1A/B.^386,387^ The transcription coactivators YAP and PDZ binding motif (TAZ) serve as pivotal downstream effectors of this pathway.^386,387^ Hippo signaling lacks specialized receptors or extracellular ligands upstream and relies on other signaling pathways to regulate its activation.^386,387^ Nonetheless, upon activation, the Hippo-YAP signaling pathway inhibits downstream YAP nuclear translocation and transcriptional activity, leading to YAP degradation.^386,387^

After myocardial I/R injury, the activation of Wnt/β-catenin signaling can promote YAP1 transcription, thereby inhibiting Hippo-YAP signaling and subsequently suppressing cell hypertrophy induced by myocardial I/R injury^142^ (Fig. 9c). In addition, inhibition of the Hippo-YAP signaling pathway can also attenuate myocardial fibrosis, initiating heart regeneration and restoration of MI-induced heart damage.^379,388^ In post-MI neonatal hearts, YAP activates the non-canonical Wnt signaling pathway in cardiomyocytes via the downstream target gene *Wls*, and inhibits the expression of *NFAT, Col1a1, Postn*, and *Fn1*, thereby suppressing cardiac fibroblast proliferation, collagen formation, and the inflammatory response.^388^ Additionally, YAP/TAZ participates in the composition of destruction complexes within the canonical Wnt signaling pathway, modulating the accumulation or degradation of β-catenin in response to Wnt signaling activation or inhibition, respectively.^389^ Overall, this provides insight into the mechanism underlying Wnt/Hippo-YAP signal crosstalk.

Targeting the crosstalk between the Hippo/YAP and Wnt/β-catenin signaling pathways is a potential therapeutic approach for myocardial and cerebral I/R injury. For instance, exogenous melatonin has been shown to regulate the expression of *miR-143-3p*, activating downstream target genes *Yap* and *Ctnnd1*. Upregulated Ctnnd1 may activate the Wnt/β-catenin signaling pathway, promoting the formation of β-catenin and Yap complexes and enhancing cardiomyocytes proliferation post-MI.^390^ Further, crosstalk between YAP and the non-canonical Wnt signaling pathway (Wnt/ROR1/2) can delay the process of cardiac fibrosis during MI.^388^ Amani et al. designed and synthesized anti-transferrin receptor monoclonal antibody (OX26)-polyethylene glycolated selenium nanoparticles that promoted Wnt3a/β-catenin activation. When combined with YAP1, the nanoparticles can enhance FoxO1 expression, and provide neuroprotection against oxidative stress, promoting neuronal survival after stroke.^391^

### BMP signaling

BMP, a member of the TGF-β family, is a crucial player in embryonic development^392,393^ and interacts with the Wnt signaling pathway.^394^ In brain I/R, the crosstalk between BMP and Wnt signaling regulates neurogenesis and neuronal differentiation, which is an essential process for brain injury repair. However, crosstalk between these two pathways remains unexplored in other organs.

The BMP signaling pathway can be categorized into either canonical or non-canonical pathways. In mammals, this pathway comprises over 20 ligands, 4 type I receptors, and 3 type II receptors.^395^ In the canonical pathway, BMP type II receptors bind to ligands and phosphorylate type I receptors. Phosphorylated type I receptors recruit and phosphorylate Smad1/5/8 receptors, which bind Smad4 to form complexes that translocate to the nucleus to regulate the expression of multiple downstream target genes. In the non-canonical pathway, type I receptors activate the downstream MAPK pathway, resulting in the translocation and phosphorylation of MAPK signaling proteins (p38, ERK1/2, and JNK). This phosphorylation triggers the activation of ATF2, c-JUN, and c-FOS, which controls the transcription of downstream target genes.^395,396^

The Wnt signaling pathway can promote neurogenesis and induce BMP production in differentiated neurons, facilitating astrocyte differentiation and inhibiting oligodendrocyte differentiation.^34^ BMP2, in conjunction with Wnt1 or Wnt3, helps to maintain the undifferentiated state of mouse trunk neural crest cells and promotes the formation of neural crest–derived stem cells.^397^ Neural crest cells in an undifferentiated state possess pluripotent capabilities and can differentiate into neurons, glial cells, or smooth muscle cells. Changes in the BMP signaling pathway have been observed in hypoxic-ischemic brain damage, with upregulated BMP4 signaling in perinatal hypoxic brains^398^ and neuroprotective effects of BMP7 following cerebral ischemic injury.^399^ In a rat model of hypoxic-ischemic encephalopathy, Wnt signaling promoted the differentiation of neural stem cells into neurons and oligodendrocytes by upregulating BMP2 protein expression, aiding in the repair of cerebral ischemic injury.^400^ However, activation of the Wnt/β-catenin signaling pathway can downregulate BMP4 expression, promoting striatal neurogenesis during cerebral ischemic injury.^401^ These findings suggest synergistic and antagonistic effects between the Wnt/β-catenin and BMP signaling pathways during cerebral ischemia or reperfusion (Fig. 9d).

Endoglin, a co-receptor of the TGF-β family, is indispensable during early hematopoiesis^402–404^ and can regulate BMP/Smad1 and Wnt/β-catenin signaling pathways and target Jdp2 to promote the integration of hematopoietic and cardiac progenitor cells in the heart and hematopoietic myeloid system.^405^ Furthermore, inhibition of GSK-3β by MLT and T63 activates the BMP/Smad and Wnt/β-catenin signaling pathways, initiating osteogenesis.^406^

### NMDAR-Ca^2+^-ActA signaling

NMDAR is an important ion channel for excitatory synaptic transmission, and ActA regulates synaptic plasticity through NMDAR phosphorylation activation and Ca^2+^ influx.^407^ Recent studies have highlighted the interplay between the NMDAR-Ca^2+^-ActA and Wnt/β-catenin signaling pathways in regulating synaptic plasticity during cerebral ischemia.^407^

NMDAR containing the GluN2A subunit exhibits a neuroprotective effect, while GluN2B-containing NMDAR induces excitatory neurotoxicity following ischemic I/R injury, contributing to intracellular calcium overload.^408^ In a rat model of chronic cerebral ischemia, the NMDAR-Ca^2+^-ActA and Wnt/β-catenin signaling pathways exhibited crosstalk^407^ (Fig. 9e). Furthermore, NMDAR activation-mediated calcium influx can trigger calpain activation.^409^ Activated calpain then cleaves β-catenin, allowing the resulting fragmented β-catenin to evade–degradation mediated by damaged complex and translocate into the nucleus to promote downstream *TCF* gene transcription.^409^

However, this cleaved β-catenin cannot bind to cadherin, resulting in decreased synaptic stability.^409^ In summary, the activation of the Wnt/β-catenin signaling pathway by the NMDAR-Ca^2+^-ActA signaling pathway influences synaptic transmission in two aspects, its beneficial effects on cerebral I/R remains to be determined.

Previous research has targeted NMDAR as a key receptor for the treatment of cerebral I/R injury in animal models.^410–412^ Enhancing neuronal NMDAR activity exerts a neuroprotective effect on cerebral ischemia,^411^ reducing the content of bound NMDAR and preserving the physiological function of free NMDAR. Consequently, NMDAR treatment can reduce the infarct volume of ischemic I/R injury.^412^ Taken together, the crosstalk between NMDAR-Ca^2+^-ActA and Wnt/β-catenin pathways presents a promising therapeutic approach for I/R injury.

### TLR4/TRIF signaling

The TLR family plays an integral role in the human immune system,^413^ and its pathway has been implicated in the progression of organ I/R injury.^414^ The crosstalk between the Wnt and TLR signaling pathways influences inflammation-associated cell proliferation, lung cancer cell proliferation, invasion, and metastasis.^415,416^ Recent findings have reported the presence of Wnt signaling during liver I/R injury, demonstrating crosstalk between Wnt and TLR signaling pathways.

The TLR family consists of 10 members and is involved in inflammation. The TLR signaling pathway comprises MyD88-dependent and MyD88-independent pathways, specific to TLR3 and TLR4, respectively. In MyD88-dependent pathway, MyD88 recruits IL-1 receptor-associated kinase to its C-terminal TIR structural domain, leading to phosphorylation of IL-1 receptor-associated kinase and activation of JNK and NF-κB.^413^

Tong et al. established that when receiving liver I/R, the serum transaminase levels of C1 wild-type mice were significantly elevated when treated with recombinant WISP1 protein. However, in WISP1-treated TLR4 knockout or junction-induced interferon β (TRIF) knockout mice, these levels were not elevated. The authors concluded that WISP1 causes liver I/R damage in mice through TLR4/TRIF signaling, and both factors play a synergistic role in hepatic IRI^414^ (Fig. 9f).

Furthermore, Mark et al. identified the expression of Wnt5a within human and mouse atherosclerotic lesions using apolipoprotein e-deficient mice and concluded that activation of the TLR-4 signaling cascade induces Wnt5a expression, and the crosstalk between TLR-4 and atypical Wnt family members, including Wnt5a, synergistically contributes to atherosclerosis.^417^ Additionally, a TLR4/AKT pathway that mediates Wnt5a expression has also been identified in human dental pulp stem cells.^418^

Although the therapeutic role of targeting TLR4/TRIF crosstalk through the Wnt signaling pathway has been explored in cardiac injury and neurological disorders, research regarding this crosstalk mechanism in I/R injury remains limited. Salwa et al. determined that the flavonoid baicalin reduced cardiac TLR4 overexpression, downregulated NF-κB expression, inhibited inflammation, attenuated cardiac fibrosis, and exerted cardioprotective effects in a doxorubicin-induced cardiotoxicity mouse model of heart injury.^419^ In addition, baicalin ameliorated dobiezosin-induced cardiomyopathy by significantly reducing cardiac levels of the secretory protein DKK1, upregulating Wnt/β-catenin activity, and attenuating cardiac inflammation and oxidative stress. The Wnt/β-catenin pathway has been suggested to play an antagonistic role with TLR4 in cardiac injury.^419^ Alternatively, REM sleep deprivation activated the TLR4/NF-κB pathway and inhibited the Wnt/β-catenin pathway in rats, resulting in neuronal damage and cognitive dysfunction in the CA1 region of the hippocampus and cerebral cortex. Nonetheless, oral administration of asparagine reversed this effect in rats and ameliorated the associated sleep disturbance and cognitive dysfunction induced by REM sleep deprivation.^420^

### HGF/c-Met signaling

The HGF/c-Met signaling pathway is associated with cell proliferation, survival, apoptosis, migration, and embryogenesis^421^ and regulates cell proliferation and fibrotic processes during renal I/R through GSK3-mediated crosstalk with the Wnt/β-catenin signaling pathway. However, data on whether this crosstalk mechanism is involved in heart, brain, or liver I/R injury remain limited.

HGF consists of α and β chains that are bound via disulfide bonds, while c-Met is a MET family RYK. The activity of c-Met is initiated when the β chain of HGF binds to the Sema region of c-Met. Activated c-Met undergoes dimerization and autophosphorylation, leading to the recruitment of intracellular growth factor receptor binding protein 2 and PI3K, as well as activation of downstream signaling pathways.^422,423^

Crosstalk occurs between the HGF/c-Met and Wnt/β-catenin signaling pathways during renal I/R (Fig. 9g). HGF binds to the c-Met receptor and activates downstream Akt, which promotes GSK-3β amino-terminal Ser9 phosphorylation, inhibits GSK-3β activity, and activates the Wnt/β-catenin signaling pathway. Combined treatment with anti-TNF-α and HGF has been demonstrated to attenuate renal fibrosis caused by renal I/R injury in mice.^424^ After renal I/R injury, the remaining renal tubular epithelial cells are crucial for repairing the injured renal units.^425^ Koraishy et al. reported that, in early renal ischemia, activated HGF promotes LRP5/6 phosphorylation in dedifferentiated tubular epithelial cells.^426^ The phosphorylated LRP5/6 disrupts the destruction complex, leading to the accumulation and nuclear translocation of β-catenin, thereby activating the Wnt/β-catenin signaling pathway and exerting anti-apoptotic effects. This phosphorylation of LRP5/6 depends on the activation of the c-Met receptor, which recruits active GSK3 to LRP5/6, rather than being stimulated by Wnt protein.^426^ Further, β-catenin regulates HGF secretion, and the crosstalk between Wnt/β-catenin and HGF/c-Met signaling pathways enhances intercellular communication. Renal tubular epithelial cell–derived Wnt proteins communicate with mesenchymal fibroblasts via paracrine secretion.^427^ AKI increases renal tubular epithelial cell–derived Wnt proteins, which target mesenchymal fibroblasts and activate the Wnt/β-catenin signaling pathway. Inhibition of Wnt/β-catenin signaling induces HGF secretion from mesenchymal fibroblasts following renal I/R injury. HGF activates the HGF/c-Met signaling pathway, promoting renal tubular cell survival and proliferation.^428^ This suggests a negative regulatory effect of β-catenin on HGF, and the crosstalk between these two signaling pathways facilitates communication between renal tubular cells and mesenchymal fibroblasts, ultimately exacerbating AKI following renal I/R injury.

The HGF/c-Met signaling pathway also plays an important role in the treatment of cerebral ischemia.^426^ In a mouse stroke model, intrastriatal injection of HGF solution promoted cell proliferation and inactivate MMP activity, maintaining BBB integrity.^429^ It has also been found to protect against apoptosis and autophagy in rats with transient MCAO^430^ and stimulate neurogenesis in neural stem cells of the SVZ when directly injected into the cerebral parenchyma.^431^ Furthermore, BB3, a small molecule with HGF-like activity, can cross the BBB and improve neurological function after ischemic stroke by stimulating the HGF pathway.^432^ In renal I/R injury experiments, knockdown of β-catenin in fibroblasts activated the HGF/c-Met signaling pathway and promoted renal tubular cell proliferation.^428^ Based on these findings and the identified crosstalk mechanisms between HGF/c-Met and Wnt signaling, targeting crosstalk signaling pathways may hold promise as an effective therapeutic approach for organ I/R injury.

## Therapeutic strategies

I/R injury is a leading cause of death in ischemic diseases, posing a significant challenge for clinicians in developing effective treatment strategies. This complication can arise in both surgical and non-surgical scenarios, and despite the development of various therapeutic approaches such as antiplatelet and antithrombotic agents, their effectiveness in reducing I/R injury remains limited. Therefore, there is a pressing need for novel treatment strategies to address ischemic diseases more effectively. The Wnt signaling pathway, along with its interplay with other signaling pathways, emerges as a critical regulator of the occurrence and progression of I/R injury. Consequently, targeting this signaling network holds promise as an innovative therapeutic strategy for this condition.

### Potential therapeutic strategies targeting Wnt signaling in I/R injury

Several therapeutic approaches focusing on Wnt signaling, including cell and exosome therapy, gene therapy, protein therapy, and drug therapy, have shown promising prospects for clinical application. Table 1 summarizes the therapeutic strategies targeting Wnt signaling for the treatment of I/R injury.

**Table 1:** Therapeutic strategies targeting Wnt signaling for the treatment of I/R injury

Therapeutic strategy	Target pathways	Strategy/Molecular/ Drugs	Organ	Effects	References
Cell therapy	Active Wnt/β-catenin pathway	Exosomes isolated from adipose-derived mesenchymal stem cells, ADMSCs-EX	Heart	Up-regulate Wnt3a; Inhibiting apoptosis	^95^
		Oligodendrocyte precursor cell transplantation	Brain	Promoting angiogenesis; Repair BBB integrity	^239^
		Transplanted adipose stem cell exosomes (ADSCs-Exo)	Liver	Inhibit NF-κB pathway; Reducing pyroptosis of damaged liver	^296^
Gene therapy	Active Wnt/β-catenin pathway	Down-regulated miR-148b	Heart	Up-regulate Wnt1; Inhibiting apoptosis and oxidative damage	^93^
		Up-regulation of LncRNA AZIN1-AS1	Heart	LncRNA AZIN1-AS1/miR-6838-5p active Wnt3a /β-catenin; Inhibiting apoptosis	^94^
		Nur77 gene ablation	Brain	Inhibiting mitochondrial fragmentation	^176^
		Overexpression of LncRNA NEAT1	Brain	Stable Wnt3a; Inhibiting apoptosis	^174^
		Down-regulation of lncRNA MEG	Brain	Promoting neurogenesis	^232,252^
Molecular therapy	Inhibit Wnt non-canonical pathways	Up-regulation of Sfrp5	Heart	Inhibit Wnt5a/JNK and Wnt/PCP; Inhibiting apoptosis and inflammation	^96–99,102^
Medication	Active Wnt/β-catenin pathway	A polypeptide of tuna stem protein, APTBP	Heart	Inhibiting apoptosis	^127,128^
		Phyllanthus emblica (*P. emblica*)	Heart	Active PI3K/Akt/GSK3β/β-catenin; Inhibiting apoptosis and collagen fibrosis	^334^
		CHIR99021 (GSK3β inhibitor)	Heart	Active Hippo pathway; Inhibiting apoptosis and cell hypertrophy	^137^
		Isoflurane	Brain	Inhibiting apoptosis	^165^
		XQ-1H; gastrodin	Brain	Inhibiting apoptosis; Promoting neurogenesis	^175,252^
		Peroxynitrite; Mallotus oblongifolius;ellagic acid	Brain	Promoting neurogenesis	^221,229,230^
		Curcumin	Brain	Promoting neurogenesis; Inhibiting apoptosis; Relieving inflammation	^208,211,231^
		TWS119	Brain	Repairing BBB; Reducing neuroinflammation	^209^
		TWS119	Brain	Inhibit Notch; Inhibiting apoptosis	^318^
		Quercetin	Brain	Repairing BBB	^246,254^
		Human serum albumin	Brain	Reducing oxidative stress	^212^
		Galangin	Brain	nhibit HIF-1α/VEGF; Improving the neurovascular unit microenvironment	^343^
		Dexmedetomidine	Brain	Active PI3K/AKT; Reducing cerebral infarct volume; Promoting neuronal survival	^333,334^
		Wnt agonist (a synthetic pyrimidine)	Kidney	Inhibiting inflammatory response and oxidative stress	^293^
		Minocycline	Liver	Reducing oxidative stress; Inhibiting the release of proinflammatory cytokines	^287^
		Agmatine	Liver	Promoting cell proliferation; Reducing inflammation and apoptosis	^282^
		Losartan	Liver	Up-regulate HIF-1α and Wnt/β-catenin signaling pathways; Up-regulate IL-6, IFN-γ, and Wnt3a; Reducing liver blood flow; Reducing liver congestion, vacuolization and necrosis	^285^
	Inhibit Wnt/β-catenin pathway	Dexamethasone, Dex	Heart	MiR-208b-3p/Med13/Wnt/β-catenin; LncRNA CCAT1/miR-8063/Wnt/β-catenin; Inhibit Wnt3a and Wnt5a; Inhibitingapoptosis	^333,334^
		Baicalin	Heart	Reducing oxidative damage to cardiomyocytes	^414^
		Huoxin pill	Heart	Inhibit NF-κB; Inhibiting inflammation	^107,366^
		Melatonin	Kidney	Improving renal fibrosis	^385^
	Inhibit Wnt non- canonical pathways	Curcumin	Brain	Inhibit Wnt/PCP; Promoting neurogenesis; Inhibiting apoptosis; Relieving inflammation	^211,231^

#### Preclinical Studies on therapeutic strategies targeting Wnt in I/R injury

A recent clinical trial utilizing the GSK-3 inhibitor Tideglusib has demonstrated the feasibility of targeting GSK-3 in human diseases.^433,434^ Furthermore, lithium, another GSK-3 inhibitor used for bipolar disorder, has shown no significant adverse effects on the heart.^435,436^ Wen-Bin Fu et al. have extensively reviewed the therapeutic effects of Wnt pathway inhibitor in the treatment of MI.^437^ Several inhibitors, including pyrvinium,^438^ UM206,^439^ ICG-001,^440^ Wnt-974,^128^ CGX1321,^441^ and GNF-6231^91^ have proven to be safe in clinical trials and exhibit potential for MI treatment. Additionally, Novel Wnt pathway inhibitors like Cardionogen^442^ and IWR1,^443^ have also been developed. Moreover, Wnt pathway inhibitors have recently garnered attention as potential anti-tumor medicine and are currently being investigated in ongoing clinical trials. These advancements have sparked further interest in exploring the effects of Wnt pathway inhibitors^61^ on organ I/R injury^437^; however, the development of novel Wnt pathway inhibitors with minimal clinical toxicity and unique effects on heart remains of utmost importance.

### Enhancing therapeutic strategies by targeting the Wnt/crosstalk signaling pathway in I/R injury

In the pursuit of overcoming I/R injuries, clinicians are currently utilizing preconditioning and postconditioning approaches. These strategies have shown promising therapeutic effects by specifically targeting the intricate network of signaling pathways implicated in I/R pathology.

#### Preconditioning

Preconditioning methods have been employed to mitigate I/R injury encompass (IPC), remote ischemic preconditioning (RIPC), and pharmacological preconditioning.^444^ IPC treatment effectively triggers the production and release of various endogenous ligands, including adenosine,^445^ bradykinin,^446,447^ opioids,^448^ norepinephrine,^449^ and acetylcholine.^450^ These pharmacological pretreatment strategies have demonstrated efficacy in preventing I/R injury.^444^ Upon administration of IPC, the respective ligand binds to its receptor^431^ subsequently initiating downstream signaling cascades.^443,444^ The Preconditioning treatment has been shown to activate various potential pathways, including the Wnt/β-catenin,^451–458^ PI3K,^459^ Akt,^460^ PKC, eNOS,^461^ GSK-3β phosphorylation, ERK1/2, p38, MAPK,^462^ and JAK-STAT3 signaling. Previous research suggested that the cardioprotective effects can be achieved through targeting of GSK-3β *via* Wnt signaling pathway.^463,464^ Correa-Costa et al. postulated that Wnt signaling might play a crucial role in protection against the renal I/R injury when the ischemic IPC treatment strategy is applied.^465^

Currently, RIPC is considered a safe and highly appealing conditioning technique for minimizing additional damage caused by ischemic^466^ and has been shown to upregulate VEGF expression, followed by activation of endothelial transcription factor Id1, Wnt2, and β-catenin expression.^467^ This suggests that RIPC exerts its protective effects on organs, at least in part, by modulating the Wnt signaling pathway. Furthermore, RIPC decreases myocardial I/R injury by activating the JAK/STAT pathway through the involvement unacylated ghrelin.^468^ However, a drawback of both IPC and RIPC treatment strategies is that they must be administered prior to the onset of an ischemic event, which can be unpredictable in clinical scenarios. Consequently, researchers have developed postconditioning treatment strategies to overcome this limitation.

#### Postconditioning

Postconditioning encompasses different techniques, namely ischemic postconditioning (IPOSTC), remote ischemic postconditioning (RIPOSTC), and pharmacological postconditioning (PPC). IPOSTC, a relatively recent method, can be applied during reperfusion initiation to reduce infarct size.^469–471^ The organ protection effects of IPOSTC are mediated by the activation of network transduction pathways, including the PI3K/Akt, PI3K/Akt/eNOS/NO, MAPK, NF-κB, Gluk2/PSD95/MLK3/MKK7/JNK3, JAK2/STAT3, eNOS, MEK1/2/Erk1/2, GSK-3β, β-catenin, reperfusion injury salvage kinase, and Akt/pkB pathways.^472–482^ RIPOSTC is a technique that entails subjecting a distant organ to brief I/R at the onset of reperfusion in the affected organ.^483^ RIPOSTC is more applicable in clinical settings as it can be performed on non-vital organs, minimizing the risk of damage to the affected organ caused by reperfusion therapy following ischemia. This clinical approach is also suitable for long-term rehabilitation.^484,485^ RIPOSTC exerts its organ protection effects through the activation of network signaling pathways, including the eNOS, PI3K, Akt, GSK-3β, and T-LAK-cell-originated protein kinase pathways. It also enhances endogenous antioxidant enzyme activity, and inhibit δ-PKC.^486–490^ PPC is a therapeutic strategy applied after a severe ischemic event or at the onset of reperfusion. Various medications, including morphine, propofol, and sufentanil, have been used in clinical practice as part of PPC to prevent I/R injury. Previous studies have shown that morphine can activate Wnt/β-catenin signaling^491–493^; propofol has a therapeutic effect against esophageal cancer,^494^ gastric cancer,^495^ hepatocellular carcinoma,^496^ and colon cancer^497^ by blocking Wnt/β-catenin signaling; Sufentanil inhibits the proliferation of lung cancer cells by suppressing Wnt/β-catenin signaling.^498^ During PPC, Wnt/β-catenin signaling may be one of the molecular pathways involved in protecting organs from further damage.

Preconditioning and postconditioning strategies have demonstrated efficacy in preventing I/R in injury in clinical settings. However, the clinical feasibility of preconditioning is limited, while postconditioning holds more promise for application in clinical practice. Therefore, future research should prioritize investigating the molecular mechanisms underlying postconditioning to translate these findings into effective clinical strategies.

## Discussion and perspectives

The Wnt signaling pathway encompasses various signaling branches, among which Wnt/β-catenin, Wnt/PCP, and Wnt/Ca^2+^ are the principal pathways implicated in organ I/R injury. Through a comprehensive analysis of available literature, it has been established that both canonical and non-canonical Wnt signaling pathways exhibit consistent patterns during the process of ischemia and reperfusion. Specifically, the canonical Wnt/β-catenin pathway is activated during the ischemic phase. This activation of the canonical pathway plays a beneficial role in injured organs through various processes such as inflammation, ECM remodeling, angiogenesis, fibrosis, and nerve regeneration. Within different organs,^49,120–122,134,135,146,499–501^ it serves as a compensatory protective response aimed at mitigating damage caused by ischemia and promoting the repair of resulting injuries. However, when these mechanisms become decompensated, corresponding pathological processes occur. In vitro experiments showed that neuronal OGD/R treatment can activate the Wnt/β-catenin signaling pathway to inhibit apoptosis and improve neuronal survival,^171^ further confirm the idea that activation of Wnt/β-catenin function to organ protect. Studies have demonstrated that as ischemia progresses, that Wnt/β-catenin signaling is inhibited in the heart, brain, and liver during the reperfusion phase. This inhibition leads to detrimental processes including apoptosis, ferroptosis, inflammation, inhibition of nerve regeneration and angiogenesis, and disruption of BBB.^174–176,178,198,214,215,233,244,247,253,348,400^ These pathological events contribute to organ damage promotion. In summary, the activation of the canonical Wnt/β-catenin pathway during ischemia serves as a protective mechanism, while its inhibition during reperfusion contributes to organ damage. On the other hand, activation of the non-canonical Wnt pathway contributes to organ damage during both ischemia and reperfusion.^151,172^ In the case of myocardial ischemia, the Wnt/PCP signaling pathway is activated, while during cerebral ischemia, the Wnt/Ca^2+^ signaling pathway is activated.^151,172^

Conflicting reports exist regarding the role of Wnt/β-catenin signaling during ischemia and reperfusion phases. Some studies showed that Wnt/β-catenin was inhibited in the heart during ischemia phase, contrary to its activation,^78,502^ while other studies reported that Wnt/β-catenin was activated during the reperfusion phase in renal I/R injury, instead of being inhibited.^263–268^ These contradictory findings may be attributed to the varying effects of Wnt/β-catenin within different organs or different cell types within the same organ. Further investigation is required to gain a comprehensive understanding of the role of Wnt/β-catenin in different contexts. In summary, our review supports the notion that activation of the canonical Wnt/β-catenin pathway serves as a protective factor, while non-canonical pathways act as mechanisms that promote organ damage. However, the precise role of Wnt/β-catenin signaling during ischemia and reperfusion requires further investigation to reconcile the conflicting reports and establish a clearer understanding of its implications in I/R injury. Detailed information on the effects of Wnt signaling pathways during I/R injury in four different organs are listed in Table 2.

**Table 2:** Effects of Wnt signaling pathways during I/R injury in four different organs

Phase	Wnt signaling pathway	Activity	Organ	Effect	References
Ischemia	Wnt/β-catenin	Activated	Heart	Promoting inflammation, ECM remodeling, angiogenesis, fibrosis	^115–117,129,130,138–141^
			Brain	Promoting neurogenesis (in vitro)	^221^
			Kidney	Promoting apoptosis and oxidative stress	^256^
		Inhibited	Heart	Promoting oxidative stress	^78,126^
			Brain	Promoting apoptosis, ferroptosis, inflammation, inhibiting neurogenesis, inhibiting angiogenesis, destroying BBB	^169–171,173,193,209,210,228,239,242,248,343,395^
	Wnt/PCP	Activated	Heart	Promoting inflammation, cell hypertrophy	^146^
	Wnt/Ca^2+^	Activated	Brain	Promoting apoptosis (in vitro)	^167^
Reperfusion	Wnt/β-catenin	Inhibited	Heart	Promoting apoptosis inflammation, cell hypertrophy	^93–95,120,137^
			Brain	Promoting apoptosis, inflammation, oxidative stress, inhibiting neurogenesis, inhibiting angiogenesis, destroying BBB	^10,164,165,174,175,207,232,244,246^
			Liver	Promoting apoptosis, oxidative stress, inflammation, inhibiting cell proliferation	^267–271,283,285,293,296^
		Activated	Brain	Inhibiting apoptosis (in vitro)	^166^
			Kidney	Inhibiting apoptosis, promoting apoptosis (in vitro), mitophagy, cell autophagy, cell aging and renal fibrosis	^25,252,253,258–263^
	Wnt/PCP	Activated	Heart	Promoting apoptosis, inflammation, angiogenesis	^50,102^
			Brain	Promote apoptosis, inflammation	^29,208^
	Wnt/Ca^2+^	Activated	Heart	Promoting apoptosis	^103^
			Kidney	Promoting renal fibrosis	^264^
			Liver	Promoting apoptosis	^103,272,273^

The Wnt signaling pathways exhibit crosstalk with a various key signaling pathways, forming a network that play a broad role in the regulation of I/R injury. Besides the mentioned crosstalk signaling pathways, we hypothesize that other signaling pathways may also be involved in this mechanism, among them, the Rho/Rho-associated protein kinase,^503,504^ MAPK/ERK,^505^ JAK/STAT,^468,506^ Nrf2,^507^ and AMPK^508^ signaling pathways deserve further investigation.

Co-targeting Wnt signaling pathways and their crosstalk signaling pathways presents a promising therapeutic strategy for I/R injury. In recent years, TCM has emerged as a research focus for treating organ I/R injury due to its potential therapeutic effects, minimal side effects, and promising outcomes in clinical rehabilitation. Given the diverse components of TCM, its therapeutic targets often involve multiple signaling pathways. Studies by Zhao et al.^509^ and Li et al.^510^ reported the beneficial effects of TCM components such as Astragalus,^511,512^ Salvia Miltiorrhiza,^513,514^ Angelica Sinensis,^515,516^ Harpagide,^517^ Icariin,^518^ pachymic acid.^519^ Zhao et al.^509^ and Li et al.^510^ have shown that treatment with Astragalus,^511,512^ Salvia Miltiorrhiza,^513,514^ Angelica Sinensis,^515,516^ Harpagide,^517^ Icariin,^518^ pachymic acid,^519^ and Lycopene^197,520,521^ confer therapeutic effects against I/R injury in the heart, brain, and other organs. Moreover, Zhao et al.^509^ reported that TCM like Tricin,^522^ Platycodin D,^523^ Baicalein,^524^ Lupeol,^525^ Paeoniflorin,^526^ and Bauhinia Championii^527^ could activate the PI3K/Akt signaling pathway and mitigate brain I/R injury. Additionally, these TCM treatments have also been found to modulate the activity of the Wnt/β-catenin signaling pathway in different developmental or pathological contexts.^528–537^ Moreover, TCM have shown activation of PI3K/Akt,^522–526^ NF-κB,^538–540^ HIF-1α,^541^ and Notch signaling pathways^542^ during organ injury treatment, indicating their potential to alleviate I/R injury by targeting multiple signaling pathways. Exercise-based cardiac rehabilitation has demonstrated numerous benefits for patients with cardiac disease, including a reduced risk of MI.^543^ Our previous research has shown that programmed exercise can inhibit pathological ventricular hypertrophy and myocardial fibrosis gene expression through the suppression of PKC-α/NFAT signaling in a mouse model.^544^ Furthermore, in an arrhythmogenic cardiomyopathy mouse model, treadmill exercise restored transcriptional levels of most differentially expressed genes and improved dysfunctional biological pathways associated with EMT, inflammation, and Wnt signaling, indicating a connection between exercise and signaling modulation.^545^ Therefore, we propose that a combined therapy involving targeting related network signaling pathways and exercise intervention may benefit the recovery of patients with cardiac or other organ I/R injury.

Overall, this comprehensive review of the Wnt/crosstalk signaling pathways network implicated in organ I/R injury underscores the need for novel treatment strategies in I/R injury.

Currently, most therapeutic interventions target individual signaling pathways, neglecting the complexity of the network. Therefore, future research efforts should be directed toward developing approaches that modulate this network signaling system as a cohesive unit. Such a comprehensive approach holds immense clinical potential and has the capacity to significantly enhance patient survival rates and improve their quality of life. Understanding and targeting the interconnected signaling pathways will help to facilitate the development of effective and holistic therapeutic interventions in the management of organ I/R injury.

### Supplementary information

Author Checklist
