# Supplementary material for: Ischemia-reperfusion injury: molecular mechanisms and therapeutic targets
Source: Signal Transduct Target Ther. 2024 Jan 8;9:12. doi: 10.1038/s41392-023-01688-x (PMC10772178; doi:10.1038/s41392-023-01688-x)
Supplement: Supplementary file 1 — Author Checklist [file 41392_2023_1688_MOESM1_ESM.pdf]

Author Checklist before final submission for Review (STTT)

- ☑ **Authorship:** no more than **10** authors, no more than **3** corresponding authors and no more than **5** co-first authors.
- ☑ The **title** is suggested to be in no more than **15** words.
- ☑ The **title** should avoid use of jargon, abbreviations, and question.
- ☑ **Abstract** should be unstructured and **200-250** words.
- ☑ **Affiliations including ones in Hong Kong, Macau/Macao and Taiwan/Taipei**: pay attention that the last **must be** "Hong Kong, China" or "Macau/Macao, China" or "Taiwan/Taipei, China" (**Caution: The Comma is NECESSARY!**).
- ☑ Please upload the "**Change of Authorship Request Form**" when submitting the revision if any changes concerning the authorship (including but not limited to: **removal** and/or **addition** of any authors, **change of authors' order**, etc.) are made. You can download this form at: https://resource-cms.springernature.com/springer-cms/rest/v1/content/7454878/data/v5
- ☑ **Image** resolution of at least 300 dpi at publication size.
- ☑ **Figures** divided into parts should be labelled with a **lower-case**, **boldface** 'a', 'b', etc. in the top left-hand corner.
- ☑ **Figures** use the same typeface (Arial or Helvetica) for all figures. Use symbol font for Greek letters.
- ☑ **Figures** are best prepared at the size you would expect them to appear in print. At this size, the optimum font size is 8pt and no lines should be thinner than 0.25 pt (0.09 mm).
- ☑ **Reference style** (All authors should be listed for papers with up to five authors; for papers with more than five authors, the first only should be listed, followed by et al. Abbreviations for titles of medical periodicals should conform to those used in the latest edition of Index Medicus. The first and last page numbers for each reference should be provided.)

☑ Please add the statement "All authors have read and approved the article" in **Author Contributions** part.

☑ When describing COVID-19-associated background and results, please **avoid using** the words such as "Wuhan virus/strain", "Hubei", "spread from China", etc.

☑ **Figures** (or parts of figures) generated from online tools (e.g., **BioRender, etc**) or including online picture resources (e.g. from **SMART - Servier Medical ART, etc**) should be clearly indicated by **citing the original websites in the figure legend** or following the requirements mentioned in the original websites.

☑ **No Graphic Abstract.**

☑ **World map** is strongly **discouraged** from being used in figures.

☑ **Figure style** (please refer to **Example 1 and 2**)

Example 1:

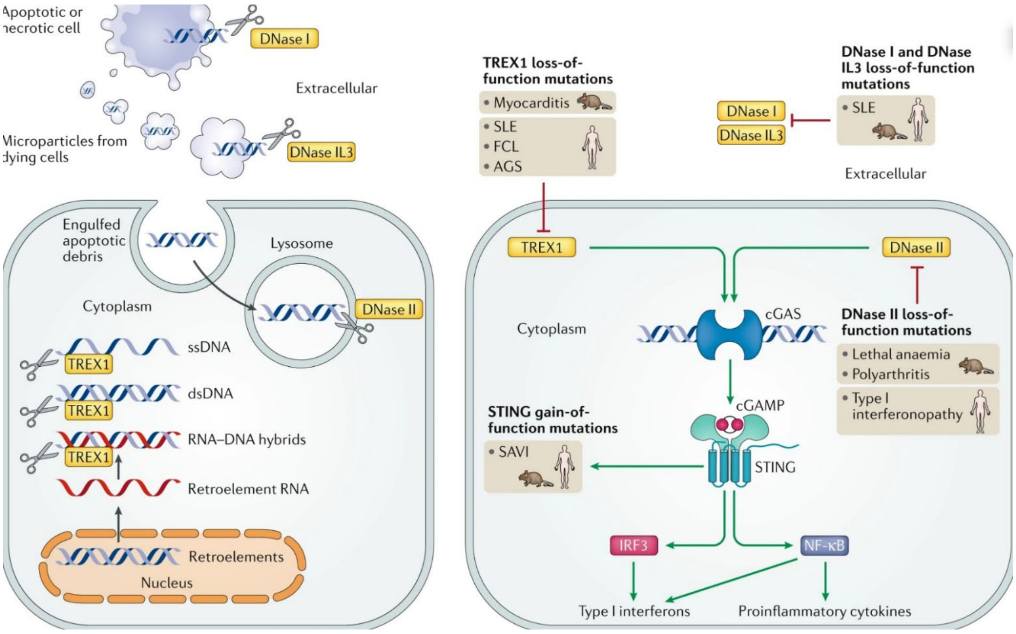

Example 2:

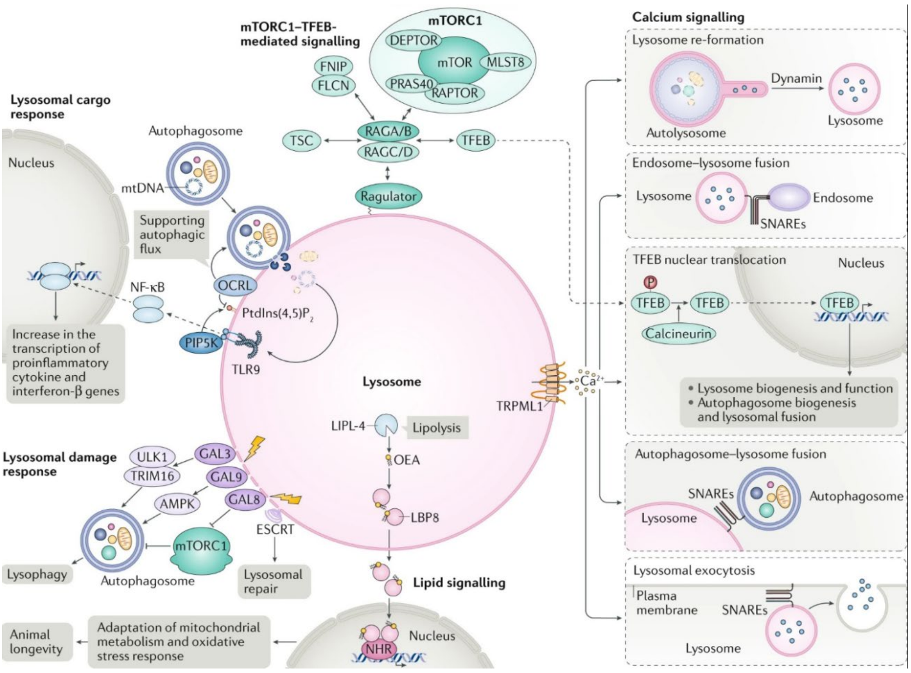

mTORC1–TFEB-mediated signalling
mTORC1
DEPTOR
mTOR
MLST8
PRAS40
RAPTOR
FNIP
FLCN
TSC
RAGA/B
RAGC/D
TFEB
Ragulator
Lysosomal cargo response
Autophagosome
Nucleus
mtDNA
Supporting autophagic flux
OCRL
NF-κB
PtdIns(4,5)P2
PIP5K
TLR9
Increase in the transcription of proinflammatory cytokine and interferon-β genes
Lysosome
LIPL-4
Lipolysis
OEA
LBP8
TRPML1
Ca2+
Lysosomal damage response
ULK1
TRIM16
GAL3
GAL9
GAL8
AMPK
ESCRT
mTORC1
Lysophagy
Autophagosome
Lysosomal repair
Lipid signalling
Nucleus
NHR
Animal longevity
Adaptation of mitochondrial metabolism and oxidative stress response
Calcium signalling
Lysosome re-formation
Dynamin
Autolysosome
Lysosome
Endosome–lysosome fusion
Lysosome
Endosome
SNAREs
TFEB nuclear translocation
Nucleus
P
TFEB
TFEB
TFEB
Calcineurin
• Lysosome biogenesis and function
• Autophagosome biogenesis and lysosomal fusion
Autophagosome–lysosome fusion
SNAREs
Autophagosome
Lysosome
Lysosomal exocytosis
Plasma membrane
SNAREs
Lysosome
